# A pH-Sensitive Fluorescent Chemosensor Turn-On Based in a Salen Iron (III) Complex: Synthesis, Photophysical Properties, and Live-Cell Imaging Application

**DOI:** 10.3390/molecules28217237

**Published:** 2023-10-24

**Authors:** Nicole Nilo, Mauricio Reyna-Jeldes, Alejandra A. Covarrubias, Claudio Coddou, Vania Artigas, Mauricio Fuentealba, Luis F. Aguilar, Marianela Saldías, Marco Mellado

**Affiliations:** 1 Instituto de Química, Facultad de Ciencias, Pontificia Universidad Católica de Valparaíso, Valparaíso 2373223, Chile; nicole.nilo@pucv.cl (N.N.); vania.artigas@pucv.cl (V.A.); mauricio.fuentealba@pucv.cl (M.F.); luis.aguilar@pucv.cl (L.F.A.); 2 Laboratory of Cancer Biology, Department of Oncology, Old Road Campus Research Building, University of Oxford, Oxford OX3 7DQ, UK; mauricio.reynajeldes@oncology.ox.ac.uk; 3 Laboratorio de Señalización Purinérgica, Departamento de Ciencias Biomédicas, Facultad de Medicina, Universidad Católica del Norte, Coquimbo 1781421, Chile; alejandra.covarrubias@ucn.cl (A.A.C.); ccoddou@ucn.cl (C.C.); 4 Millennium Nucleus for the Study of Pain (MiNuSPain), Santiago 8330025, Chile; 5 Facultad de Ciencias Agropecuarias, Universidad del Alba, La Serena 1700000, Chile; 6 Núcleo para el Estudio del Cáncer a Nivel Básico, Aplicado, y Clínico, Universidad Católica del Norte, Coquimbo 1781421, Chile; 7 Instituto de Investigación y Postgrado, Facultad de Ciencias de la Salud, Universidad Central de Chile, Santiago 8330507, Chile; marianela.saldias@ucentral.cl

**Keywords:** pH chemosensor, fluorescence turn-on, live cell imaging, gastric cancer, metabolic sensor, lactate

## Abstract

pH regulation is essential to allow normal cell function, and their imbalance is associated with different pathologic situations, including cancer. In this study, we present the synthesis of 2-(((2-aminoethyl)imino)methyl)phenol (HL1) and the iron (III) complex (Fe(L1)_2_Br, (**C1**)), confirmed by X-ray diffraction analysis. The absorption and emission properties of complex **C1** were assessed in the presence and absence of different physiologically relevant analytes, finding a fluorescent turn-on when OH^−^ was added. So, we determined the limit of detection (LOD = 3.97 × 10^−9^ M), stoichiometry (1:1), and association constant (K_as_ = 5.86 × 10^3^ M^−1^). Using DFT calculations, we proposed a spontaneous decomposition mechanism for **C1**. After characterization, complex **C1** was evaluated as an intracellular pH chemosensor on the human primary gastric adenocarcinoma (AGS) and non-tumoral gastric epithelia (GES-1) cell lines, finding fluorescent signal activation in the latter when compared to AGS cells due to the lower intracellular pH of AGS cells caused by the increased metabolic rate. However, when complex **C1** was used on metastatic cancer cell lines (MKN-45 and MKN-74), a fluorescent turn-on was observed in both cell lines because the intracellular lactate amount increased. Our results could provide insights about the application of complex **C1** as a metabolic probe to be used in cancer cell imaging.

## 1. Introduction

It is well known that intra- and pericellular pH are essential for normal cell function. Maintenance of intracellular pH is essential for establishing the outcome of many cellular processes, including cell growth and differentiation, cell metabolism, and protein synthesis [1]. For example, cancer cell metabolism is characterized by enhanced glucose use and uptake [2], decreasing intracellular pH when compared to non-tumoral cells [3]. In this sense, cancer cells create a reversed pH gradient with their pericellular pH being more acidic than normal cells, associated with abnormal glycolytic activity and other metabolic alterations that lead to an increase in pyruvate conversion into lactate instead of citrate [4], acidifying the extracellular medium. Low extracellular pH in cancer cells plays a significant role in drug inactivation or reduced uptake and the promotion of invasiveness and metastatic potential [3]. Due to the essential role of pH in cellular fate, the ability to monitor and visualize pH values and distribution using non-invasive probes is in high demand. Therefore, fluorescent and colorimetric sensors have attracted widespread attention for their potential use in the detection of numerous environmental and biologically important species because of their operational simplicity, low cost, fast response, and, in the best of cases, high sensitivity, selectivity, and satisfactory detection limits [5].

**Figure 1:**
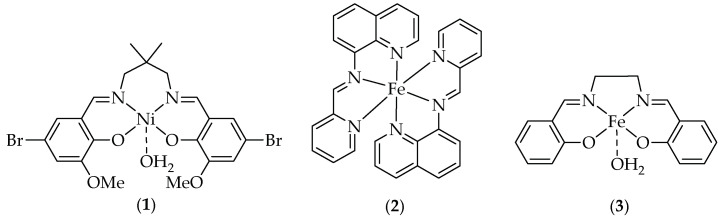
Metal complexes with chemosensor properties.

Fluorescent probes are widely utilized as selective analyte-binding devices, often with the capability of reversible binding. For instance, coumarin, thiophene-based Schiff bases, and azo dyes are commonly employed to detect physiologically relevant or potentially harmful metal centers [6,7,8,9,10], as well as toxic organic residues originating from industries such as textiles, agriculture, and pharmaceuticals [11,12]. Furthermore, fluorescent probes can be utilized to detect specific anions [6,13,14,15] within living tissues. These probes play a crucial role in the detection and monitoring of analytes, offering valuable insights into various biological and environmental processes. Salen-type Schiff base chemosensors, as depicted in Figure 1, are particularly noteworthy for their suitability as molecular frameworks for sensing applications. Many of these chemosensors form coordination compounds with first-row transition metal centers, resulting in luminescent materials [16]. This feature contributes to their usefulness as luminescent probes in sensing applications, allowing for the detection and monitoring of analytes through changes in their emission properties. Chemosensors based on coordination metal compounds have demonstrated their effectiveness in detecting highly toxic anions. These chemosensors have been successfully employed in the detection of hazardous species such as cyanide, halogens [17], and/or toxic industrial gases [14]. Furthermore, they have shown promise in distinguishing between various basic anions in aqueous environments and even within living systems. The inherent stability of coordination metal compounds in the presence of analytes within their coordination spheres contributes to their robust performance as chemosensors. This stability has been demonstrated by N. Mohan et al., who reported on a fluorescent probe based on Ni(II)-salen (**1**). This probe enables the detection of metallic analytes such as Al(III) through a transmetalation process [18]. Similarly, J. Yu et al. developed a turn-off–on fluorescence chemosensor utilizing an Fe(III)-salen complex (**2**), which exhibits a redox response for the detection of hydrazine [19].

For instance, a recent study focused on the development of a reaction-based chemical reagent, Fe(III)-salen (**3**), specifically designed for sensing mitochondrial inorganic pyrophosphate (PPi) in living cells [20]. PPi is a byproduct involved in biosynthetic reactions and serves as a diagnostic marker for various diseases [21]. The Fe(III)-salen reagent demonstrates the potential to selectively detect and monitor PPi levels within mitochondria, providing valuable insights into cellular processes and potential disease states.

The objective of this study is to investigate a novel fluorescent sensing system utilizing Fe(III)-salen for the detection of alkaline pH. Furthermore, this study aims to evaluate the fluorescent differentiation capabilities of this system between gastric cancer cells (AGS cell line) and non-tumor cells (GES-1 cell line). As an extension of the chemosensing application in cancer cells, the assessment was extended to metastatic tumor cell lines, specifically MKN-45 and MKN-74. However, it was observed that the altered metabolic cycle of these cell lines resulted in an increase in lactate levels and an alkaline intracellular environment.

## 2. Results

### 2.1. Chemistry

**Scheme 1:**
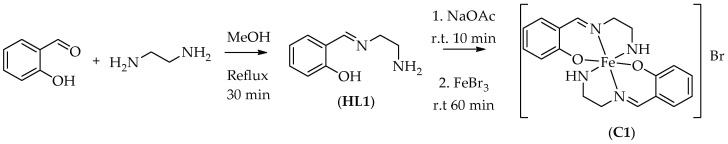
Synthesis of ligand **HL1** and the Fe(III) complex (**C1**).

The ligand 2-(((2-aminoethyl)imino)methyl)phenol (**HL1**) was synthesized by reflux for 30 min using salicylaldehyde and ethylenediamine to avoid the formation of the tetradentade ligand. The resulting yellow solution was dried and quickly analyzed by spectroscopic techniques. The infrared spectrum of **HL1** exhibited a strong intensity absorption stretching band at ν = 1635 cm^−1^ that was attributed to C=N vibration, which is evidence of imine condensation (Figure S1) and is concordant with a previous report [22]. Then, the ligand solution (**HL1**), 1.0 mol equivalent of Na_2_CO_3_, and 0.5 mol equivalent of FeBr_3_ to ensure the stoichiometry 2:1 (ligand: Fe(III)) were mixed and refluxed for 1 h, forming a dark-red solution (Scheme 1). This solution was filtered and slowly evaporated at room temperature, yielding the iron (III) complex (**C1**) as a dark, red-colored solid. Using infrared and Raman spectroscopic techniques, the iron (III) complex (**C1**) was analyzed. In both spectrums, the **C1** compound showed the same band at ν = 606–608 cm^−1^ and ν = 447–445 cm^−1^, which was attributed to Fe-O and Fe-N vibrations, respectively (Figure S2).

**Figure 2:**
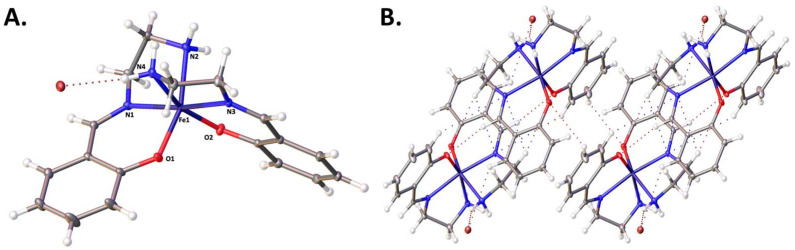
Crystal structure of iron (III) complex **C1**. (**A**) Structure of complex with ellipsoids drawn at 30% probability. (**B**) Packing diagram of complex (**C1**) showing intermolecular N_imine_⋯H (blue), O⋯H (red), and Br⋯H (dark red) hydrogen bonds along the plane [010].

Single crystals of iron (III) complex (**C1**) were grown by diffusing methane, and X-ray diffraction analysis indicated that **C1** crystalizes in a triclinic P-1, with one molecular entity in the asymmetric unit (Z′ = 1). The N_2_O ligands coordinate meridionally to the Fe(III) metal center (configuration *mer*, Figure 2A). Table S1 exhibits selected bond distances and angles for the coordination sphere. Specifically, it is described as a distorted octahedron [23] with Σ = 79.6°, Θ = 226.1° at 170 K, and α = 100.8°, which indicates that the octahedron is highly distorted. Finally, crystal packing (Figure 2B) exhibited principal intermolecular N_imine_⋯H, O⋯H, and Br⋯H hydrogen bonds along the plane [010].

### 2.2. Photophysical Characterization and Chemosensing Capacity Assessment

The photophysical properties of the iron (III) complex (**C1**) were assessed from a stock dissolution at a concentration of 1.0 × 10^−2^ M in DMSO and stored in the dark at −4 °C [24,25,26]. From the stock solution, absorption properties were measured using dilutions in acetonitrile, obtaining a maximum absorption wavelength at λ_Abs_ = 330 nm and an absorption coefficient ε = 5358.5 M^−1^·cm^−1^ corresponding to the π → π* transition, while the maximum absorption wavelength at λ_Abs_ = 504 nm with an absorption coefficient ε = 1812.3 M^−1^·cm^−1^ could be assigned to the charge-transfer transition metal → ligand (Figure S3) [22]. Subsequently, the behavior of the iron (III) complex (**C1**) in the presence of different inorganic salts was assessed (Figure S4), showing a slight change in the maximum absorption wavelength (λ_Abs_ = 330 nm) in all the samples tested. The case of the hydroxide (OH^−^), metabisulfite (S_2_O_5_^−2^), and nitrate (NO_3_^−^) treatments caused a hyperchromic effect on the metal–ligand transition wavelength. Interestingly, only the OH^−^ sample showed a new electronic transition band at λ_Abs_ = 370 nm (Figure S4), confirming a change in the electronic states of the complex (**C1**) [27]. Due to these changes in the absorption spectra after addition of NaOH to the iron (III) complex (**C1**), the stability of this interaction was evaluated, showing high responses in a short time (3 min), which were stable up to 30 min in the absorption band λ_Abs_ = 386 nm (Figure S5).

**Figure 3:**
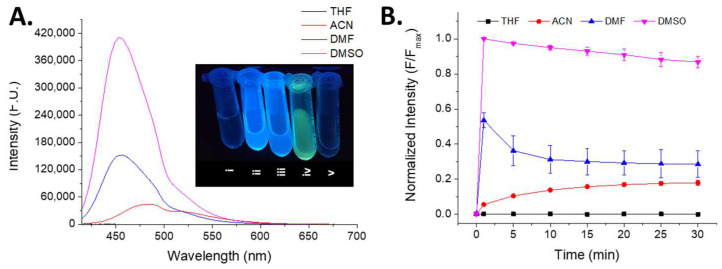
Emission response of the iron (III) complex (**C1**) after the addition of NaOH. (**A**) Spectrum overlapping and emission intensity change at various water rates. (**B**) Intensity emission behavior using different DMSO:Water mixes at λ_Em_ maximum in each solvent. λ_Ex_ = 399 nm; λ_Em_ 0% H_2_O = 456 nm; λ_Em_ 10% H_2_O = 458 nm; λ_Em_ 20% H_2_O = 480 nm; λ_Em_ 30% H_2_O = 487 nm; λ_Em_ 40% H_2_O = 488 nm; λ_Em_ 50% H_2_O = 488 nm; λ_Em_ 100% H_2_O = 489 nm; C_C1_ = 20 µM; C_NaOH_ = 10 mol equivalents; solvent = DMSO. Image code: i: Iron (III) complex (**C1**) dissolved in DMSO. ii: Iron (III) complex (**C1**) dissolved in DMSO with NaOH 10 molar equivalents. iii: Iron (III) complex (**C1**) dissolved in DMSO:H_2_O: 9:1 with NaOH 10 molar equivalents. iv: Iron (III) complex (**C1**) dissolved in DMSO:H_2_O: 8:2 with NaOH molar equivalents. v: Iron (III) complex (**C1**) dissolved in DMSO:H_2_O: 7:3 with NaOH 10 molar equivalents. vi: Iron (III) complex (**C1**) dissolved in DMSO:H_2_O: 6:4 with NaOH 10 molar equivalents. vii: Iron (III) complex (**C1**) dissolved in DMSO:H_2_O: 5:5 with NaOH 10 molar equivalents. viii: Iron (III) complex (**C1**) dissolved in H_2_O with NaOH 10 molar equivalents.

Because of their potential application in biological sciences, the emission property was assessed considering the absence and presence of several of the previously mentioned analytes. Initially, dissolution of the iron (III) complex (**C1**) did not show fluorescent properties, with this effect explained by the paramagnetic properties of the iron (III) ion [28,29]. Moreover, the change in emission properties after the addition of analytes was evaluated, finding a selective fluorescence turn-on after NaOH was added to the iron (III) complex (**C1**) solution. Therefore, changes in the emission frequency of complex **C1** using different solvents and their influence on the recognition of NaOH were measured (Figure S6), finding the highest emission rates using DMSO. In addition, by increasing the amounts of water interacting with NaOH from DMSO:H_2_O: 10:0 to DMSO:H_2_O: 9:1, fluorescence intensity decayed by approximately 40% (Figure 3). This trend was also observed when the amount of water was increased from 20% to 50%, obtaining a fluorescence intensity decay of around 80% when compared to the DMSO-only measurements.

**Figure 4:**
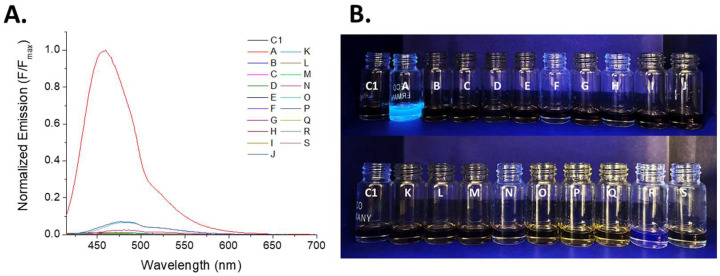
Emission response of the iron (III) complex (**C1**) after the addition of several analytes. (**A**) Overlapping emission spectrum. (**B**) Naked-eye emission change in the complex with all analytes assessed illuminated with a UV-Lamp λ = 360 nm. C_C1_ = 20 µM, C_Sample_ = 10 mol equivalents, solvent = DMSO. Sample code: A. Hydroxide (OH^−^); B. Metabisulfite (S_2_O_5_^−2^); C. Iodide (I^−^); D. Acetate (MeCO_2_^−^); E. Nitrate (NO_3_^−^); F. Sulfate (SO_4_^−2^); G. Thiosulfate (S_2_O_3_^−2^); H. Bicarbonate (HCO_3_^−^); I. Bisulfite (HSO_3_^−^); J. Iodate (IO_3_^−^); K. Cyanide (CN^−^); L. Fluoride (F^−^); M. Sulfide (S^−^); N. Carbonate (CO_3_^−2^); O. Citrate (C_6_H_5_O_7_^−3^); P. Monoacid phosphate (HPO_4_^−2^); Q. Diacid phosphate (H_2_PO_4_^−^); R. Arsenite (AsO_2_^−^); S. Tartrate (C_4_H_4_O_6_^−2^).

After characterizing the ideal conditions for iron (III) complex (**C1**) fluorescent emission, its response before and after adding the previously described analytes was studied (Figure 4), showing a specific emission fluorescence turn-on of the solution only when OH^−^ was added. Regarding the acid–base properties of all the conditions studied, only OH^−^ caused a significant change in the electronic structure of the iron (III) complex (**C1**). Therefore, the strong base property of the OH^−^ is the response to the photophysical changes, and the optical emission properties could link to the potential hydrogen transfer from the NH_2_ fragment of the iron (III) complex (**C1**) to the strong base (OH^−^), similar to what was shown in the indole core through the fluoride anion [30].

**Figure 5:**
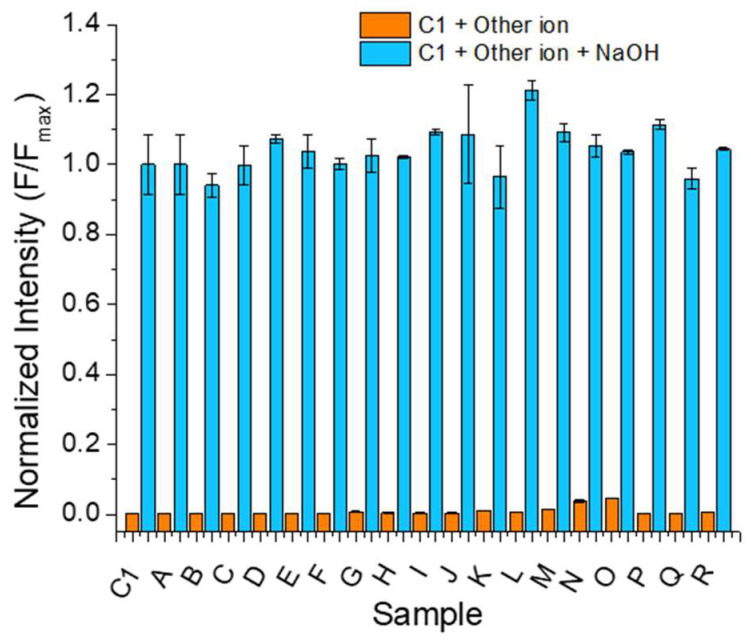
Capability of NaOH to displace the interaction of the iron (III) complex (**C1**) with other inorganic salts. Sample coding: A. Metabisulfite (S_2_O_5_^−2^), B. Iodide (I^−^), C. Acetate (MeCO_2_^−^), D. Nitrate (NO_3_^−^), E. Sulfate (SO_4_^−2^), F. Thiosulfate (S_2_O_3_^−2^), G. Bicarbonate (HCO_3_^−^), H. Bisulfite (HSO_3_^−^), I. Iodate (IO_3_^−^), J. Cyanide (CN^−^), K. Fluoride (F^−^), L. Sulfide (S^−2^), M. Carbonate (CO_3_^−2^), N. Citrate (C_6_H_5_O_7_^−3^), O. Monoacid phosphate (HPO_4_^−2^), P. Diacid phosphate (H_2_PO_4_^−^), Q. Arsenite (AsO_2_^−^), R. Tartrate (C_4_H_4_O_6_^−2^). λ_Ex_ = 399 nm and λ_Em_ = 456 nm. C_C1_= 20 µM, C_Sample_ = 10 mol equivalents, solvent = DMSO. Inorganic salts = 10 molar equivalents, incubation time = 1 min, *n* = 3.

To further investigate the selectivity of the Fe(III)-salen complex in the fluorescence turn-on process, the potential interference of various analytes was assessed, as depicted in Figure 5. In this experimental setup, different interferents, such as metabisulfite, iodide, acetate, and others, were introduced and mixed with the iron (III) complex (**C1**). After an incubation period of 3 min, the fluorescence emission was measured. Immediately after measurement, NaOH was added to the previous solution, and fluorescence emission was registered again after a 3 min incubation. The hydroxide anion was capable of displacing interfering analytes like cyanide (CN^−^), nitrate (NO_3_^−^), carbonate (CO_3_^−2^), bicarbonate (HCO_3_^−^), and fluoride (F^−^) in all of the assessed samples, highlighting that the iron (III) complex (**C1**) has a preferred interaction with the strong base OH^−^. From these results, it can be reaffirmed that the alkaline strength of hydroxide can remove the hydrogen attached to the amino-terminal group (N4, see Figure 2A) of the ligand fragment, as mentioned above.

**Figure 6:**
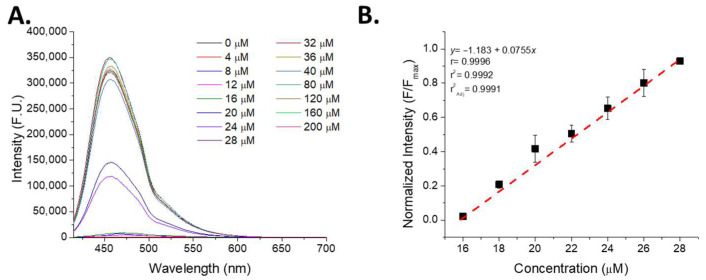
Titration of iron (III) complex (**C1**) with increasing amounts of NaOH. (**A**) Spectrum of the titration. (**B**) Linear segment of the titration. C_C1_ = 20 µM, λ_Ex_ = 399 nm; λ_Em_ = 456 nm; solvent = DMSO; incubation time = 3 min.

According to these results, the iron (III) complex (**C1**) can identify the presence of hydroxide in the absence or presence of other potential anionic interferents (Figure 4 and Figure 5). To evaluate the capacity of **C1** to interact with established amounts of hydroxide, a titration of the iron (III) complex (**C1**) was measured via their fluorescence emission responses (Figure 6A) after addition of NaOH, obtaining similar spectra along the whole titration range and a linear signal–concentration relationship between 16 and 28 µM (Figure 6B). In addition, the limit of detection (LOD) and limit of quantification (LOQ) of this interaction were calculated in the nanomolar range (LOD = 3.97 × 10^−9^ M, pH 7.009, and LOQ = 13.2 × 10^−9^ M, pH 7.029). Comparing our results with other chemosensors for alkaline pH, we found some examples of colorimetric and fluorometric sensors (displayed in Table 1), but the majority of them do not have information about their LODs, except for a 2,2-dimethyl-2,5-dihydrofuran derivative that informed a LOD of 4.08 ppb (or 102 × 10^−9^ M of NaOH equivalents), showing that the iron (III) complex (**C1**) is 25.7-fold more sensitive than this colorimetric chemosensor [31].

In parallel to the titration results, the stoichiometry of the **C1**–NaOH interaction was calculated using Job’s plot, and the constant of association was obtained using the Benesi–Hildebrand method (Figure S7), obtaining a 1:1 stoichiometry that, in contrast with the available literature, can be mechanistically explained by the transfer of the hydrogen atom from the amino to the hydroxyl group [32]. In fact, when this information was compared with the obtained crystal structure (Figure 2A), the terminal -NH_2_ of the ligand binds with bromide through one hydrogen atom, showing an important positive charge on this hydrogen atom. In addition, the association constant (K_as_) obtained for the **C1**–NaOH interaction was K_as_ = 5.86 × 10^3^ M^−1^. Comparing this value with other alkaline-sensitive compounds, our result is 4.1-fold lower than the *1H*,*3H*-benzo[*de*]isochromene derivative (Table 1, Entry 1) and 41.7-fold lower than the nitroaryl-hydrazide derivative (Table 1, Entry 3). However, despite the low K_as_ value shown for **C1**, our synthesized compound showed higher selectivity than the aforementioned chemosensors: the *1H*,*3H*-benzo[*de*]isochromene derivative can also interact with cyanide anions, while the nitroaryl-hydrazide derivative can sense fluoride, acetate, hydrogen sulfate, and also cyanide anions [33,34].

**Figure 7:**
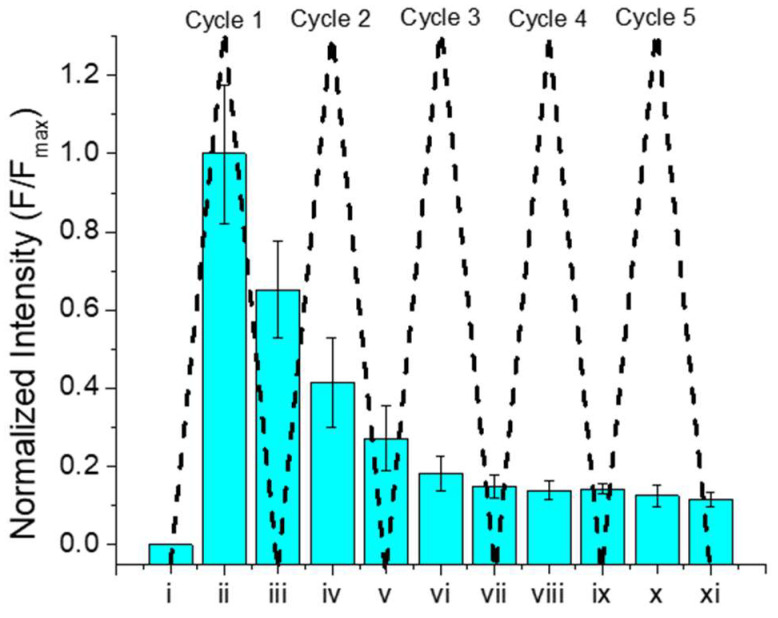
Fluorescence intensity measurements of the iron (III) complex (**C1**) after the addition of NaOH and HCl. C_C1_ = 20 µM, λ_Ex_ = 399 nm; λ_Em_ = 456 nm; solvent = DMSO; incubation time = 3 min. Sample code: i: Complex dissolved in DMSO; ii: Complex dissolved in DMSO + 1.0 equivalent mol NaOH; iii: ii + 1.0 equivalent mol HCl; iv: iii + 1.0 equivalent mol NaOH; v: iv + 1.0 equivalent mol HCl; vi: v + 1.0 equivalent mol NaOH; vii: vi + 1.0 equivalent mol HCl; viii: vii + 1.0 equivalent mol NaOH; ix: viii + 1.0 equivalent mol HCl; x: ix + 1.0 equivalent mol NaOH; xi: x + 1.0 equivalent mol HCl.

After these characterization assays, the reversibility of the fluorescence emission using the base–acid equilibrium was evaluated (Figure 7). In this figure, the complex (**C1**) dissolved in DMSO (sample i) was mixed with 1.0 equivalent mol NaOH, and, after three minutes, maximum fluorescence was measured (sample ii). Then, 1.0 mol equivalent HCl was added to the previous mixture, and fluorescence was measured again after a three-minute incubation, showing an approximate decay of 40% (sample iii) despite the neutralized NaOH that causes the turn-on of the chemosensor. This NaOH-HCl treatment cycle was repeated five times, observing a continuous decay in fluorescence intensity that stalled after cycle 2. This situation reflects that the photophysical change is irreversible, which is different from the situation reported for all the chemosensors mentioned in Table 1 (except entries 3 and 4, which were not informed). A plausible mechanism for this is the decomposition of the iron (III) complex (**C1**). More details about this mechanism are described in the following section.

### 2.3. Computational Approach

In order to sustain the experimental findings from the photophysical characterization and provide a plausible explanation for the observed phenomena, different species involved in the iron (III) complex (**C1**) and hydroxyl anion interaction using DFT-B3LYP-LanL2DZ level calculus were performed [26,37]. Initially, the structures of the iron (III) complex (**C1**) and hydroxide anion were optimized, and the electrostatic potential map (ESP) and the molecular orbitals (HOMO and LUMO) were plotted (Figure S8). In the case of the complex, the ESP map shows both aryl rings in a light-blue color, while the =N-(CH_2_)_2_-NH_2_ fragment is observed in a deep-blue color, demonstrating the positive charge of the iron (III) complex (Figure S8A). In addition, the HOMO focused on one of the salicylaldehyde fragments (Figure S8B), and the LUMO was mainly disseminated on both =N-(CH_2_)_2_-NH_2_ and the iron atom (Figure S8C). On the other hand, the ESP map of the hydroxyl anion showed an important red zone around the oxygen atom (Figure S8D), which is consistent with the HOMO plot (Figure S8E). In contrast, the LUMO focused on the hydrogen atom (Figure S8F).

**Figure 8:**
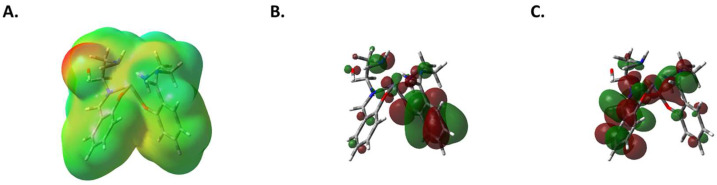
Electrostatic potential map (ESP) and molecular orbitals of the macrostructure **C1** + OH^−^. (**A**) ESP of the macrostructure. (**B**) HOMO of the macrostructure. (**C**) LUMO of the macrostructure.

**Figure 9:**
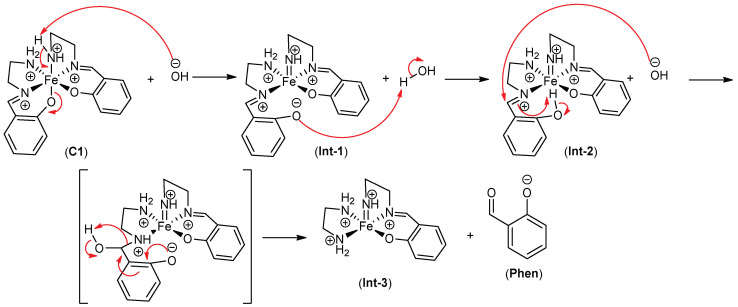
Reaction mechanism proposed for the turn-on fluorescence of the **C1** complex.

Based on the theoretical results, a hydrogen atom subtraction from the =N-(CH_2_)_2_-NH_2_ fragment of the iron (III) complex (**C1**) is plausible. In fact, due to the highly positive charge on the hydrogen atoms from the amino-terminal group (N4, Figure 2A), these atoms are labile to the hydroxyl anion presence, and this is concordant to the information shown in the hydrogen bonding of the crystal structure of the **C1** complex (Figure 2A). Then, the hydrogen transfers from the complex (**C1**) together with the hydroxyl group were calculated, showing the formation of one water molecule and complex disassembly (Figure 8). This result confirms the proposed decomposition mechanism of complex **C1**, where the initial fluorescence intensity is not recovered from the second of five NaOH-HCl treatment cycles. With all this information, a final reaction mechanism is proposed (Figure 9).

In order to increase the robustness of the proposed reaction mechanism, all the reaction steps were calculated, and the internal energy of each intermediate compound (Int) is detailed in Figure S9. Contrasting the internal energies of the **C1** complex and the proposed macrostructure **C1**+OH^−^ (Int-1, Figure 9) with the **C1** complex, there is a negative difference (ΔE = −76.996 hartree), confirming a spontaneous interaction. Moreover, the internal energy from **Int-1** to **Int-3** showed a slight decrease on each step; for example, the energy difference between **Int-1** and **Int-2** was ΔE = −0.025 hartree, while the difference between **Int-2** and **Int-3** was ΔE = −0.014 hartree. These variations in the internal energies support the spontaneous optical changes proposed in the reaction mechanism. In this way, the color and fluorescence changes to the naked eye of the salycilaldehyde and the iron (III) complex (**C1**) were tested (Figure S10), finding a similar color change in the samples using natural light as well as a similar emission fluorescence color. In addition, the proposed mechanism is similar to the one reported for chemosensor (**3**), which detects pyrophosphate via turn-on fluorescence following a disassembly approach forming the salycilaldehyde fragment [20].

To validate the proposed decomposition of the iron (III) complex (**C1**) assisted by hydroxide, the UV-Vis spectra of the macrostructure (**C1-OH**) and the other intermediates (**Int-1** to **Int-3**) were calculated and overlapped with the experimental spectra (Figure S11). This figure shows the calculated UV-Vis spectra of the macrostructure (**C1-OH**); intermediate **Int-1** has identical UV-Vis spectra. In addition, the experimental spectra contain various contributions of all intermediates calculated, among them absorption bands at 215, 254, 313, and 389 nm (Figures S12–S16, respectively). The 215 nm signal corresponds to the highly energetic absorption transitions, for example, ligand → ligand (**Int-1**, Figure S13), ligand → metal (**Int-2** and **Int-3**, Figures S14 and S15, respectively), and phenolate → benzene (**Phen**, Figure S16). In addition, the 254 nm transition only shows intermediates **Int-2** and **Int-3** (Figures S14 and S15), and the absorption bands could link to the ligand → metal transitions. In the case of the absorption transition at 389 nm, the transition phenolate → metal (**Int-3**) and the transition phenolate → benzaldehyde (**Phen**) are the most probable according to their oscillator strength and the nearest to the transition absorption band (348 nm and 360 nm, respectively; see Figures S15 and S16). Regarding the overlapping of experimental and calculated spectra (Figure S11), none of them show an absorption peak at 313 nm; however, checking thoroughly the oscillator strength of all species calculated, **Int-1** and **Int-3** showed a high oscillator (312.6 nm and 315.4 nm, respectively), and they could be associated with the experimental observations.

### 2.4. Biological Application

**Figure 10:**
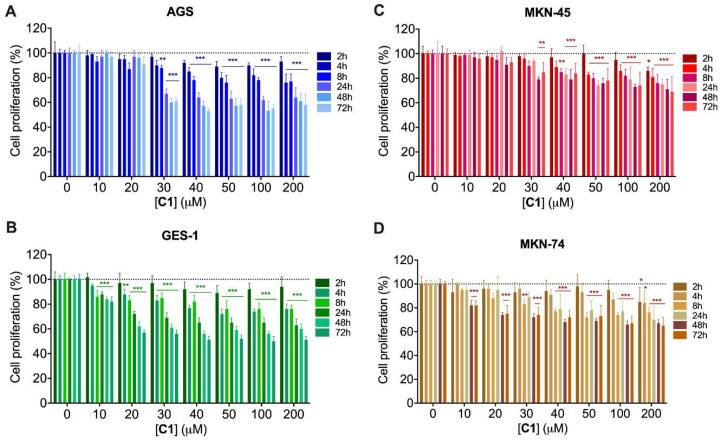
Cytotoxicity assessment of the iron (III) complex (**C1**) via Rezasurin reduction assay at different concentrations and incubation times. (**A**) AGS cell line. (**B**) GES-1 cell line. (**C**) MKN-45 cell line. (**D**) MKN-74 cell line. Significant differences were evaluated using multiple Student’s *t*-test comparisons between each treatment group and time and their corresponding probe-untreated control condition. Data are presented as means ± SD (n = 4). * *p* < 0.05, ** *p* < 0.01, *** *p* < 0.001.

In order to evaluate if **C1** is suitable for being used as an intracellular pH chemosensor, Resazurin reduction assays were carried out to observe its effects on the cell proliferation of four gastric cell lines (AGS, GES-1, MKN-45, and MKN-74) (Figure 10).

As observed in Figure 10, cell viability is not significantly compromised after an 8 h incubation with **C1** at concentrations below 30 μM. In general, 2 h incubations with compound **C1** did not have significant effects on the proliferation of the studied cell lines in the evaluated concentration range. However, longer incubation periods induced a cell line-dependent decrease in cell proliferation. For AGS and MKN-45, this reduction became significant at 30 μM (Figure 10A,C), and for GES-1 and MKN-74, these changes started at 10 μM (Figure 10B,D). Analyzing the 200 μM **C1** condition, a 31–49% reduction in cell proliferation was observed after a 72 h incubation in all the cell lines tested, with the metastatic gastric cancer cell lines MKN-45 and MKN-74 being the least affected by complex **C1**. This cytotoxic pattern is similar to previous reports for other chemosensors [38,39,40], where low concentrations and short incubation times did not induce cell damage. These results portray **C1** as a safe candidate to be evaluated as a fluorescent chemosensor capable of discriminating changes in cell metabolism by its capacity to fluctuate its fluorescent emission intensity according to the environmental pH.

**Figure 11:**
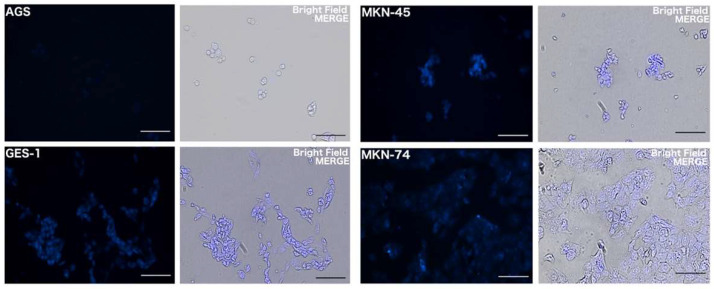
Fluorescent signal detection in gastric cell lines incubated with **C1**. Fluorescence imaging (**left**) and merged images with bright field captions (**right**) of cells after a 1 h incubation with 100 μM iron (III) complex (**C1**) on AGS, GES-1, MKN-45, and MKN-74 cells. Scale bar: 100 μm.

To evaluate this potential use, a fluorescence imaging study was carried out using the same gastric cell lines (AGS, GES-1, MKN-45, and MKN-74) submitted to a 1 h incubation with 100 µM **C1**. In the AGS cells, a weak fluorescent signal was observed in comparison to the other cell lines stabilized at extracellular pH 7.4 (Figure 11). Conversely, a bright-blue homogeneous signal was observed in GES-1 (a non-tumoral cell line), attributing this difference to a highly aerobic metabolism of AGS cells that has an impact reducing intracellular pH [41]. In addition, MKN-45 and MKN-74 cells also showed a blue fluorescent signal, with MKN-45 being the condition that displayed the most intense signal (Figure 11). Regarding AGS cells, our findings showed an apparent intracellular pH lower than 6.9 by the faint fluorescence intensity observed by microscopy, which is similar to the results obtained for an amphiphilic fluorescent probe used in AGS cells equilibrated at pH 7.0 [42]. This phenomenon is also supported by previous report on AGS, MKN-28, MKN-45, and SNU-1 gastric cancer cell lines, who, are capable to tolerate and maintain cell viability under acidic extracellular pH conditions through their H^+^ extrusion capacity via H^+^/K^+^ ATPase overexpression [43]. However, the extent of this pump overexpression could be different in each cell line. In addition to this, MKN-45 and MKN-74 also have increased protein levels of the Na^+^/H^+^ exchanger pump, which is associated with even higher intracellular pH levels, which can, in turn, enhance glycolytic metabolism [42,44].

**Figure 12:**
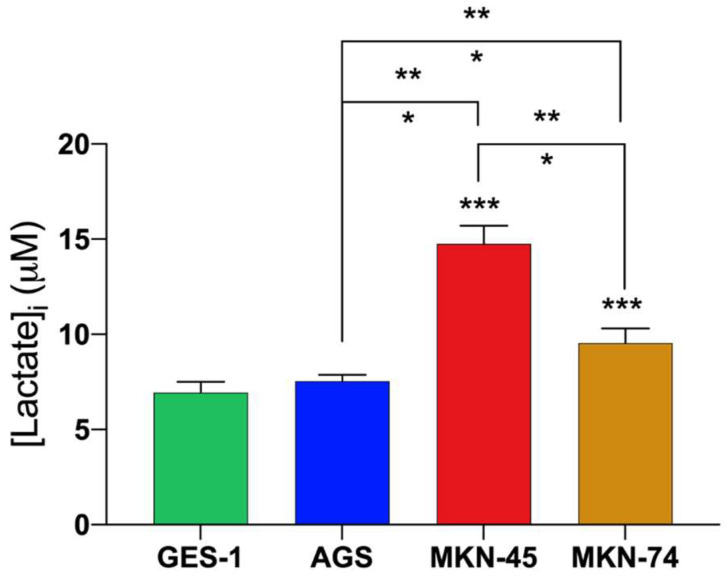
Intracellular lactate concentrations in GES-1, AGS, MKN-45, and MKN-74 cells. Mean lactate levels in these cell lines were significant when evaluated using one-way ANOVA and Tukey’s multiple comparison test. Data are presented as means ± SD (*n* = 9). * *p* < 0.05, ** *p* < 0.01, *** *p* < 0.001.

As mentioned above, metastatic tumor cells MKN-45 and MKN-74 can enhance aerobic glycolytic pathways, leading to intracellular lactate accumulation [45,46,47]. This condition is promoted in cancer cells to avoid apoptotic signaling and immune recognition [48,49]. Thus, fluctuations in intracellular pH could be explained, in part, by changes in the intracellular lactate concentration. For this reason, intracellular lactate levels were quantified (Figure 12), finding a significant intracellular lactate increase in the metastatic gastric cancer cell lines MKN-45 and MKN-74 when compared to the non-tumoral gastric cell line GES-1 and the primary gastric adenocarcinoma cell line AGS (*p* < 0.001). The highest intracellular lactate concentration was found in MKN-45 cells, which corresponds to a poorly differentiated (high-grade) metastatic (advanced-stage) gastric adenocarcinoma cell line. The opposite situation is observed in AGS, a primary gastric adenocarcinoma cell line (early stage and grade), where their intracellular lactate levels are similar to those observed for GES-1. Considering this information, the intense fluorescent signal observed for the MKN-45 and MKN-74 cells can be explained by the increased intracellular lactate levels that increase intracellular pH.

## 3. Materials and Methods

### 3.1. Instrumentation

Infrared spectra were recorded using a Jasco FT-IR 4600 instrument (Tokyo, Japan). Recorded range for IR spectra was 400–4000 cm^−1^, and samples were examined using KBr disks. Raman spectra were obtained using a RAMAN/SNOM Alpha 300 WITec confocal microscope (Grimbergen, Belgium) with a laser line at 785 nm. The recorded range for the RAMAN spectra was 100–1650 cm^−1^, and samples were examined directly in solid state on the sample holder at room temperature.

### 3.2. Chemical

The following reagents were all purchased from Sigma-Aldrich/Merck and used without further purification: salicylaldehyde ( reagent grade, 98%), ethylenediamine ( reagent grade, ≥99%), triethylamine ( reagent grade, 99%), anhydrous iron (III) bromide ( reagent grade, ≥99.99%), and anhydrous methanol (reagent grade, 99.8%). In addition, the following salts used for the chemosensor assessment were purchased from Sigma-Aldrich/Merck all of them with reagent grade >99%: NaOH, Na_2_S_2_O_5_, KI, Na_2_CH_3_CO_2_, NaNO_3_, Na_2_SO_4_, Na_2_S_2_O_3_, NaHCO_3_, NaHSO_3_, NaIO_3_, NaCN, NaF, Na_2_S, Na_2_CO_3_, Na_3_C_6_H_5_O_7_, Na_2_HPO_4_, NaH_2_PO_4_, NaAsO_2_, and Na_2_C_4_H_4_O_6_.

### 3.3. Synthetic Procedure

#### 3.3.1. Synthesis of 2-(((2-aminoethyl)imino)methyl)phenol (**HL1**)

The ligand was prepared according to a method described previously [22] by refluxing salicylaldehyde (0.192 mL, 1.83 mmol) and ethylenediamine (0.184 mL, 2.75 mmol) in absolute methanol (20 mL) for 30 min. The resulting yellow solution of HL1 was subsequently used for complex formation. Yield: 60 %. Color: yellow oil. FT-IR ν cm^−1^: 3430(br) ν(O-H), 2933(m) ν(CH_3_), 2898(m) ν(αCH_2_), 2863(α’CH_2_), 2723(w) ν(O-H···N), 1635(s) ν(C=N).

#### 3.3.2. Synthesis of *bis*-2-(((2-aminoethyl)imino)methyl)phenol Iron (III) Complex [Fe(L1)2]Br (**C1**)

The new complex was synthesized at room temperature by using tridentate NNO-donor Schiff HL1 (400 mg, 2.44 mmol) and sodium acetate (200 mg, 2.44 mmol) for 10 min, after adding iron (III) bromide (360 mg, 1.22 mmol) for 1 h. The color of the solution turned dark red and was then filtered. By slow evaporation of the solution at room temperature, dark-red-colored X-ray-quality, rectangular-shaped single crystals were obtained after two weeks. Yield: 50 %. Color: dark-red solid. M.P.: 249 °C. FT-IR ν cm^−1^: 3200(m)–3100(m) ν(N-H), 2935(m) ν(C-H, alkyl), 1626(s) ν(C=N), 774(s) ν(C-H, aromatic), 606(s) ν(Fe-O), 447(w) ν(Fe-N). RAMAN: 1626(s) ν(C=H), 608(s) ν(Fe-O), 445(w) ν(Fe-N).

### 3.4. Crystal Structure

An X-ray-suitable crystal for compound **C1** was obtained as described above. The single crystal was mounted using MiTeGen MicroMounts. ST1 shows the experimental and crystallographic data for **C1**. Intensity data were collected at room temperature on a Bruker D8 QUEST diffractometer equipped with a bi-dimensional CMOS Photon100 detector using graphite monochromated Mo-Kα radiation. The diffraction frames were integrated using the APEX3 package and were corrected for absorption with SADABS. The solution and refinement for compound **C1** were carried out with Olex2 [50]. The structure was solved by Patterson and refined with the ShelXL refinement package using least-squares minimization [51]. The complete structures were refined using the full-matrix least-squares procedure on the reflection intensities (F2). All non-hydrogens were refined with anisotropic displacement coefficients, and all hydrogen atoms were placed in idealized locations. The bond distances and angles for all compounds studied can be found in ST2. For more detailed information on the crystals, X-ray data collection, and structure solution, please refer to the Supplementary Materials and Appendix A.

### 3.5. Photophysical Properties

Molecular absorption spectra were obtained on a Shimadzu UV-mini-1240 UV-VIS spectrophotometer (Kyoto, Japan) using 10 mm path length quartz cuvettes in the range of 200–700 nm wavelengths in fast mode. Fluorescence excitation and emission spectra were obtained using a xenon arc lamp as the light source implemented on an ISS K2 Multifrequency Phase Fluorometer (Champaign, IL, USA). Fluorescence spectra were measured at 90° in an L-format using single photon counting mode. Data were acquired and analyzed by Vinci^TM^ software Version 2.4, according to our previous reports [24,25,26].

### 3.6. Computational Approach

Geometric optimizations of compounds **C1**, OH^−^, and their proposed intermediates (**Int-1** to **Int-4**) were carried out using the Gaussian 09 program [52], according to a previous report from our research group [37]. In order to obtain different chemical features, DFT-B3LYP-LanL2DZ level calculi were used in the gas phase. Optimized geometries were verified by frequency calculations without imaginary frequency on their potential energy surface. In addition, UV-Vis spectra of **C1**, **C1**-OH, and their intermediates (**Int-1** to **Int-3** and Phen) were calculated using TD-DFT-CAMB3LYP-LanL2DZ with 100 states.

The highest and lowest occupied and unoccupied molecular orbits (HOMO and LUMO, respectively) were plotted using a coarse grid (−2 points). Isovalues for both molecular orbitals were 0.02, and their densities were 0.0004. Electrostatic potential (ESP) was plotted according to total density based on a coarse grid (−2 points).

### 3.7. Biology

#### 3.7.1. Cell Culture

Cell staining experiments with the **C1** compound were performed using four different human gastric cell lines: non-tumoral gastric epithelia (GES-1), primary gastric adenocarcinoma (AGS), and moderately (MKN-74) and poorly (MKN-45) differentiated metastatic adenocarcinoma cell lines. These cells were acquired from ECACC (AGS cell number: 89090402; Porton Down, Salisbury, UK), Riken Cell Bank (MKN-45 cell number: RCB1001, and MKN-74 cell number: RCB1002; Tsukuba, Ibaraki, Japan), and Beyotime Biotechnology (GES-1 cell number: C6268; Shanghai, China). DMEM, F12K, and RPMI 1640 mediums (all from Corning, Corning, NY, USA) were used for GES-1, AGS, and MKN-45 and MKN-75 maintenance, respectively. All media were supplemented with 10% fetal bovine serum (Biological Industries, Beit HaEmek, Israel) and 1% of a 100,000 U/mL penicillin and 100,000 μg/mL streptomycin antibiotic solution (Corning, Corning, NY, USA). Cells were cultured in plastic cell culture dishes and flasks at 37 °C in a humidified atmosphere containing 5% CO_2_.

#### 3.7.2. Cytotoxic Assay

GES-1, AGS, MKN-74, and MKN-45 cells were seeded in 96-well plates (Corning, Corning, NY, USA) following a 3000 cells/well proportion to achieve 40–50% confluence after an overnight at 37 °C + 5.0% CO_2_. After cell adhesion, cells were treated with compound **C1** using a concentration range from 10 to 200 μM during 2, 4, 8, 24, 48, or 72 h. After incubation, culture medium was replaced with 70 μM Resazurin in a supplemented culture medium solution (Sigma-Aldrich, St. Louis, MO, USA). Fluorescence measurements were performed after a 6 h incubation at 37 °C + 5.0% CO_2_. Relative Fluorescent Units (RFUs) were measured using a NOVOstar Plate Reader (BMG Labtech, Ortenberg, Germany), considering 570 nm and 590 nm as excitation and emission wavelengths, respectively. Cell proliferation was calculated by comparing each experimental condition with a control condition consisting of cells incubated with supplemented medium without compound **C1**.

#### 3.7.3. Living Cell Imaging

For these experiments, 10,000 cells/well were seeded in 24-well plates with sterile 12 mm glass coverslips and incubated overnight at 37 °C + 5% CO_2_. After cell attachment, culture medium was removed from each coverslip preparation, and they were incubated with DMEM/HCO_3_^−^ pH 7.4 for 1 h at 37 °C + 5% CO_2_. After treatment, each preparation was washed twice with PBS and then permeabilized with a 0.1% Triton X-100 solution for 5 min at room temperature. Triton X-100 concentration was chosen according to previous reports focused on achieving reversible cell permeabilization without compromising cell membrane integrity [53]. After this step, cells were washed with PBS for 5 min and then incubated with 100 μM compound **C1** in DMEM/HCO_3_^−^ pH 7.4 solution for 1 h at 37 °C + 5% CO_2_. After treatment, coverslips were washed with PBS and then mounted on slides with 5 μL Fluoromount G (F4680; Sigma-Aldrich, St. Louis, MO, USA). Images were captured using an Eclipse Ts2R-FL inverted fluorescence microscope equipped with a DS-Fi3 camera (Nikon, Tokyo, Japan).

#### 3.7.4. Intracellular Lactate Measurements

Intracellular lactate concentration was measured by seeding 5000 cells/well of each gastric cell line in flat-bottom white opaque 96-well plates (136101; ThermoFisher, Waltham, MA, USA) and incubated overnight at 37 °C + 5% CO_2_ for cell attachment. The next day, culture medium was replaced, and cells were incubated again for 48 h at 37 °C + 5% CO_2_. After this incubation, culture medium was removed, and intracellular lactate measurements were performed using the Lactate-Glo Assay kit (J5022; Promega, Madison, WI, USA) following manufacturer’s instructions. To establish intracellular lactate concentrations, lactate standard curves were performed at the same time as sample measurements. Relative Light Unit (RLU) assessments were performed using a NOVOstar Plate Reader (BMG Labtech, Ortenberg, Germany).

## 4. Conclusions

In the current research, the ligand 2-(((2-aminoethyl)imino)methyl)phenol was synthesized, and, subsequently, the iron (III) complex **C1** was prepared. The identification and characterization of compound **C1** were conducted using infrared spectroscopy and X-ray diffraction. The iron (III) complex **C1** was subjected to different photophysical tests to assess its absorption and emission properties, and we found that compound **C1**’s fluorescence is turned on by adding NaOH. Through NaOH titration, the stoichiometry relationship between **C1** and OH^−^ was established as 1:1, while its limit of detection was 3.97 × 10^−9^ M. This recognition of NaOH is irreversible because of the decomposition of the iron (III) complex (**C1**), obtaining a fluorescent salicylaldehyde phenolate.

On the other hand, the **C1** complex was tested as an intracellular pH chemosensor in primary gastric adenocarcinoma cancer cells (AGSs) and compared with a non-tumoral gastric cell line (GES-1), obtaining a null optical response in the AGS cells, a phenomenon explained by their acidic intracellular pH. However, GES-1 cells showed a fluorescent signal consistent with the slightly alkaline physiological conditions. As further projections for using complex **C1** as a fluorescent probe for intracellular pH, we tested this compound in the metastatic gastric cancer cell lines MKN-45 and MKN-74, obtaining an important fluorescence turn-on in both cell lines, which could be caused by their increased intracellular pH, as reflected in the increased intracellular lactate levels of these cells when compared to AGS and GES-1 cells. The synthesis of this type of complex, its photophysical characterization and mechanism, and its properties as a viable cellular chemosensor for gastric cancer are reported for the first time. This fluorescent complex has promising properties for being used as a chemosensor for the metabolic profiling of cancer cells.

## Figures and Tables

**Table 1:** Comparison between alkaline pH chemosensors and the iron (III) complex (**C1**).

Entry	Compound	Media ^a^	Method ^b^	LOD ^c^	K_as_^d^	Ref.
1	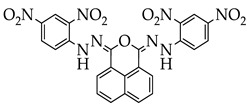	ACN:H_2_O	Colorimetric	N.R. ^e^	2.39 × 10^4^	[33]
2	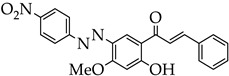	DMSO:H_2_O 9:1	Colorimetric	N.R. ^e^	N.R. ^e^	[35]
3	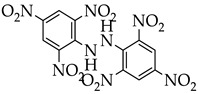	ACN	Colorimetric	N.R. ^e^	24.41 × 10^4^	[34]
4	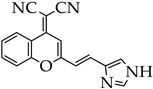	DMSO	Colorimetric	N.R. ^e^	N.R. ^e^	[32]
5	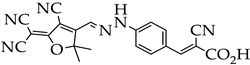	ACN	Colorimetric	4.08 ppb	N.R. ^e^	[31]
6	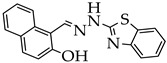	PBS:DMSO 99:1	Fluorometric	N.R. ^e^	N.R. ^e^	[5]
7	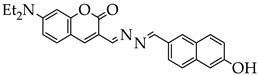	PBS:EtOH 99:1	Fluorometric	N.R. ^e^	N.R. ^e^	[36]
8	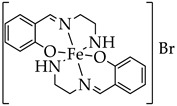 Iron (III) complex (**C1**)	DMSO	Fluorometric	0.159 ppb	5.86 × 10^3^	Our study

Media ^a^: solvent used for measurement. Method ^b^: methodology used for chemosensor evaluation. LOD ^c^: limit of detection. K_as_^d^: association constant measured by Benesi–Hildebrand method to host:guest 1:1 stoichiometry. N.R. ^e^: not reported.

## Data Availability

Not applicable.

## Supplementary Materials

The following supporting information can be downloaded at https://www.mdpi.com/article/10.3390/molecules28217237/s1, Figure S1: FT-IR spectra of ligand HL1 and the complex **C1**; Figure S2: FT-IR and Raman spectra of compound **C1**; Figure S3: Molecular absorption properties of the iron (III) complex (**C1**); Figure S4: Spectral and color change in the iron (III) complex after the interaction with several analytes; Figure S5: Kinetic profile of the interaction between the iron (III) complex (**C1**) and NaOH; Figure S6: Kinetic profile of the interaction between the iron (III) complex (**C1**) and NaOH in several solvents; Figure S7: Analytical graphs from titration of the iron (III) complex (**C1**); Figure S8: Electrostatic potential map (ESP) and molecular orbitals of the iron (III) complex (**C1**) and the hydroxyl anion; Figure S9: Internal energies of the iron (III) complex (**C1**) and the intermediates proposed in the reaction mechanism; Figure S10: Comparison of colorimetric changes between salicylaldehyde and the iron (III) complex (**C1**) after the addition of NaOH dissolution; Figure S11: Overlapping experimental UV-Vis spectra and all intermediates proposed in the reaction mechanism; Figure S12: Experimental UV-Vis spectra of complex **C1** + OH− and their calculated spectra ; Figure S13: Experimental UV-Vis spectra of complex **C1** + OH− and the calculated spectra of proposal intermediate Int-1 together with each electronic transition; Figure S14: Experimental UV-Vis spectra of complex **C1** + OH− and the calculated spectra of proposal intermediate Int-2 together with each electronic transition; Figure S15: Experimental UV-Vis spectra of complex **C1** + OH− and the calculated spectra of proposal intermediate Int-3 together with each electronic transition; Figure S16: Experimental UV-Vis spectra of complex **C1** + OH− and the calculated spectra of proposal intermediate phenolate together with each electronic transition; Table S1: Crystal data and details of structure refinement of complex **C1**; Table S2: Bond distances and selected angles in the metal coordination sphere of **C1** at 170 K; Table S3: Cartesian Coordinates of each atom from iron (III) complex (**C1**); Table S4: Cartesian Coordinates of each atom from iron (III) complex (**C1**) with analyte OH^−^.

## Sample Availability

Samples of compound **C1** are available from the authors.

## Appendix A

CCDC 2225749 contains the supplementary crystallographic data for this paper. These data can be obtained free from The Cambridge Crystallographic Data Centre via www.ccdc.cam.ac.uk/structures accessed on 29 September 2023.
