# Supplementary material for: A pH-Sensitive Fluorescent Chemosensor Turn-On Based in a Salen Iron (III) Complex: Synthesis, Photophysical Properties, and Live-Cell Imaging Application
Source: Molecules. 2023 Oct 24;28(21):7237. doi: 10.3390/molecules28217237 (PMC10647502; doi:10.3390/molecules28217237)
Supplement: Supplementary file 1 [file molecules-28-07237-s001.zip › molecules-2612624-supplementary.pdf]

SUPPLEMENTARY MATERIALS

A pH-Sensitive Fluorescent Chemosensor Turn-On Based in a Salen Iron (III) Complex: Synthesis, Photophysical Properties, and Live-Cell Imaging Application

Nicole Nilo [1], Mauricio Reyna-Jeldes [2,3], Alejandra A. Covarrubias [3,4,5], Claudio Coddou [3,4,6], Vania Artigas [1], Mauricio Fuentealba [1], Luis F. Aguilar [1], Marianela Saldías [7] and Marco Mellado [7,*]

1 Instituto de Química, Facultad de Ciencias, Pontificia Universidad Católica de Valparaíso,
Valparaíso 2373223, Chile; nicole.nilo@pucv.cl (N.N.); vania.artigas@pucv.cl (V.A.); mauricio.fuentealba@pucv.cl (M.F.); luis.aguilar@pucv.cl (L.F.A.)

2 Laboratory of Cancer Biology, Department of Oncology, Old Road Campus Research Building,
University of Oxford, Oxford OX3 7DQ, UK; mauricio.reynajeldes@oncology.ox.ac.uk

3 Laboratorio de Señalización Purinérgica, Departamento de Ciencias Biomédicas, Facultad de Medicina, Universidad Católica del Norte, Coquimbo 1781421, Chile; alejandra.covarrubias@ucn.cl (A.A.C.);
ccoddou@ucn.cl (C.C.)

4 Millennium Nucleus for the Study of Pain (MiNuSPain), Santiago 8330025, Chile

5 Facultad de Ciencias Agropecuarias, Universidad del Alba, La Serena 1700000, Chile

6 Núcleo para el Estudio del Cáncer a Nivel Básico, Aplicado, y Clínico, Universidad Católica del Norte, Coquimbo 1781421, Chile

7 Instituto de Investigación y Postgrado, Facultad de Ciencias de la Salud, Universidad Central de Chile, Santiago 8330507, Chile; marianela.saldias@ucentral.cl

* Correspondence: marco.mellado@ucentral.cl; Tel.: +56-2-2582-6567

Table of Content

1. Supplementary Figures.. 3

Figure S1: FT-IR spectra of ligand HL1 and the complex C1 3

Figure S2: FT-IR and Raman spectrum of compound C1. 3

Figure S3: Molecular absorption properties of the iron (III) complex (C1). A. UV-Vis spectra at various concentrations. B. Linear fit of absorbance and concentration at 330 nm. C. Linear fit of absorbance and concentration at 504 nm. Solvent= ACN.. 4

Figure S4: Spectral and color change of the iron (III) complex after the interaction with several analytes.. 5

Figure S5: Kinetic profile of the interaction between the iron (III)-complex (C1) and the NaOH........ 6

Figure S6: Kinetic profile of the interaction between the iron (III)-complex (C1) and the NaOH in several solvents. 6

Figure S7: Analytical graphs from titration of the iron (III)-complex (C1).. 7

Figure S8: Electrostatic potential map (ESP) and molecular orbitals of the iron (III)-complex (C1) and the hydroxyl anion. 7

Figure S9: Internal energies of the iron (III)-complex (C1) and the intermediates proposed in the reaction mechanism.......... 8

Figure S10: Comparison of the colorimetric changes between salicylaldehyde and the iron (III)-complex (C1) after and later the addition of NaOH dissolution.......... 8

Figure S11: Overlapping experimental UV-Vis spectrum and all intermediates proposed in the reaction mechanism.......... 9

Figure S12: Experimental UV-Vis spectra of complex C1 + OH- and their calculated spectra. 10

Figure S13: Experimental UV-Vis spectra of complex C1 + OH- and the calculated spectra of proposal intermediate Int-1 together to each electronic transition. 11

Figure S14: Experimental UV-Vis spectra of complex C1 + OH- and the calculated spectra of proposal intermediate Int-2 together to each electronic transition. 12

Figure S15: Experimental UV-Vis spectra of complex C1 + OH- and the calculated spectra of proposal intermediate Int-3 together to each electronic transition. 13

Figure S16: Experimental UV-Vis spectra of complex C1 + OH- and the calculated spectra of proposal intermediate phenolate together to each electronic transition. 14

3. Supplementary Tables 15

Table S1: Crystal data and details of structure refinement of complex C1. 15

Table S2: Bond diantances and selected angles in the metal coordination sphere of C1 at 170 K.......... 16

Table S3: Cartesian Coordinates of each atom from iron (III) complex (C1).......... 17

Table S4: Cartesian Coordinates of each atom from iron (III) complex (C1) with analyte OH^-.......... 19

2. Supplementary Figures

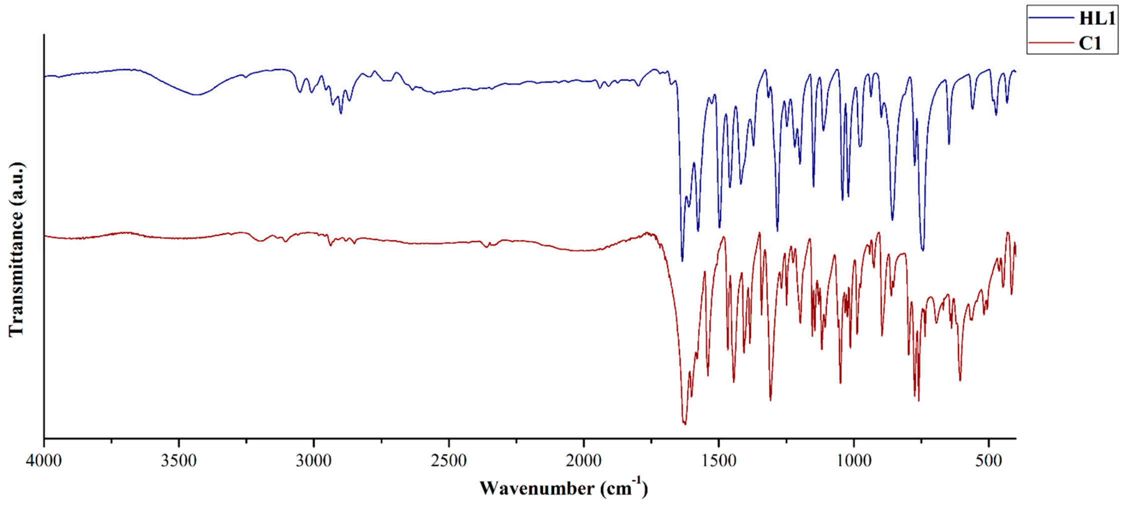

Figure S1: FT-IR spectra of ligand HL1 and complex C1

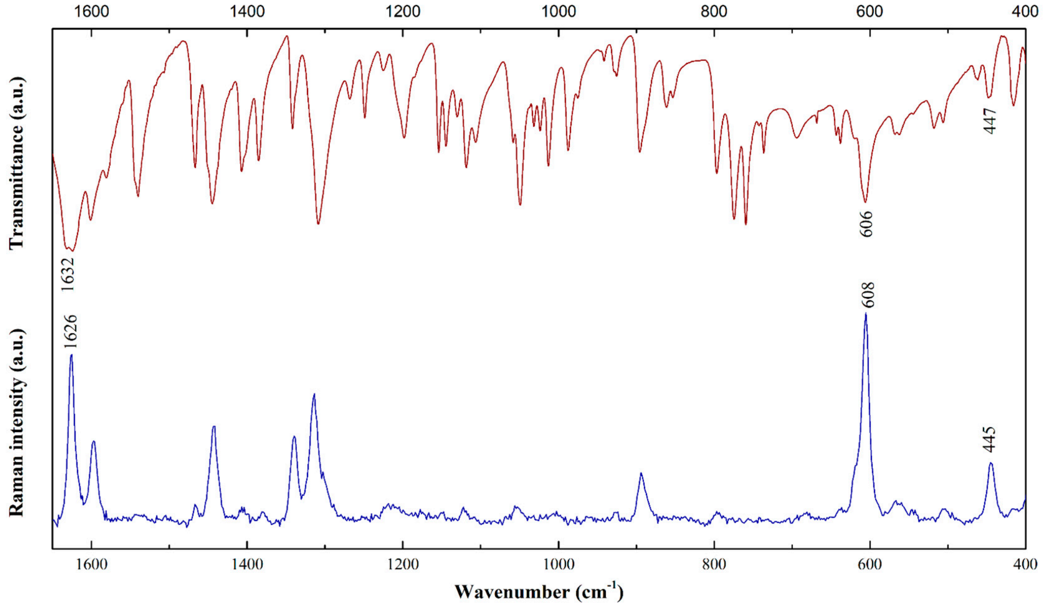

Figure S2: FT-IR and Raman spectrum of compound C1.

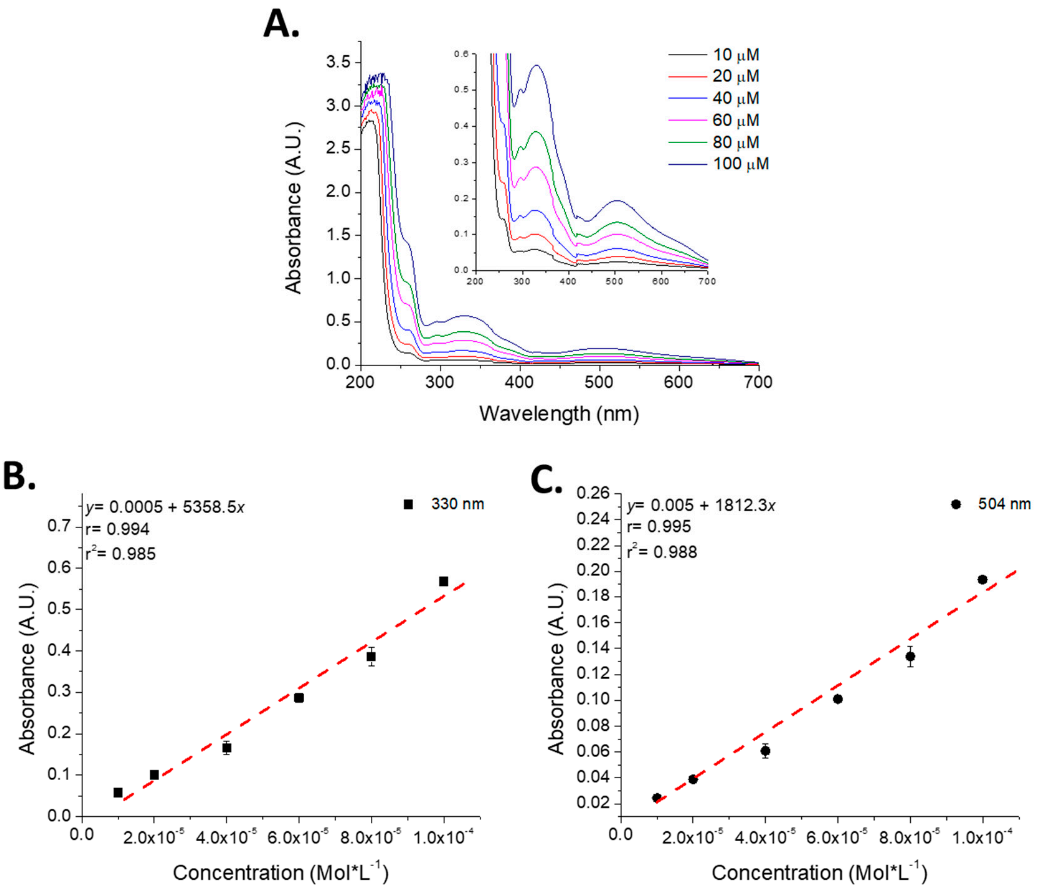

Figure S3: Molecular absorption properties of the iron (III) complex (C1). **A.** UV-Vis spectra at various concentrations. **B.** Linear fit of absorbance and concentration at 330 nm. **C.** Linear fit of absorbance and concentration at 504 nm. Solvent= ACN.

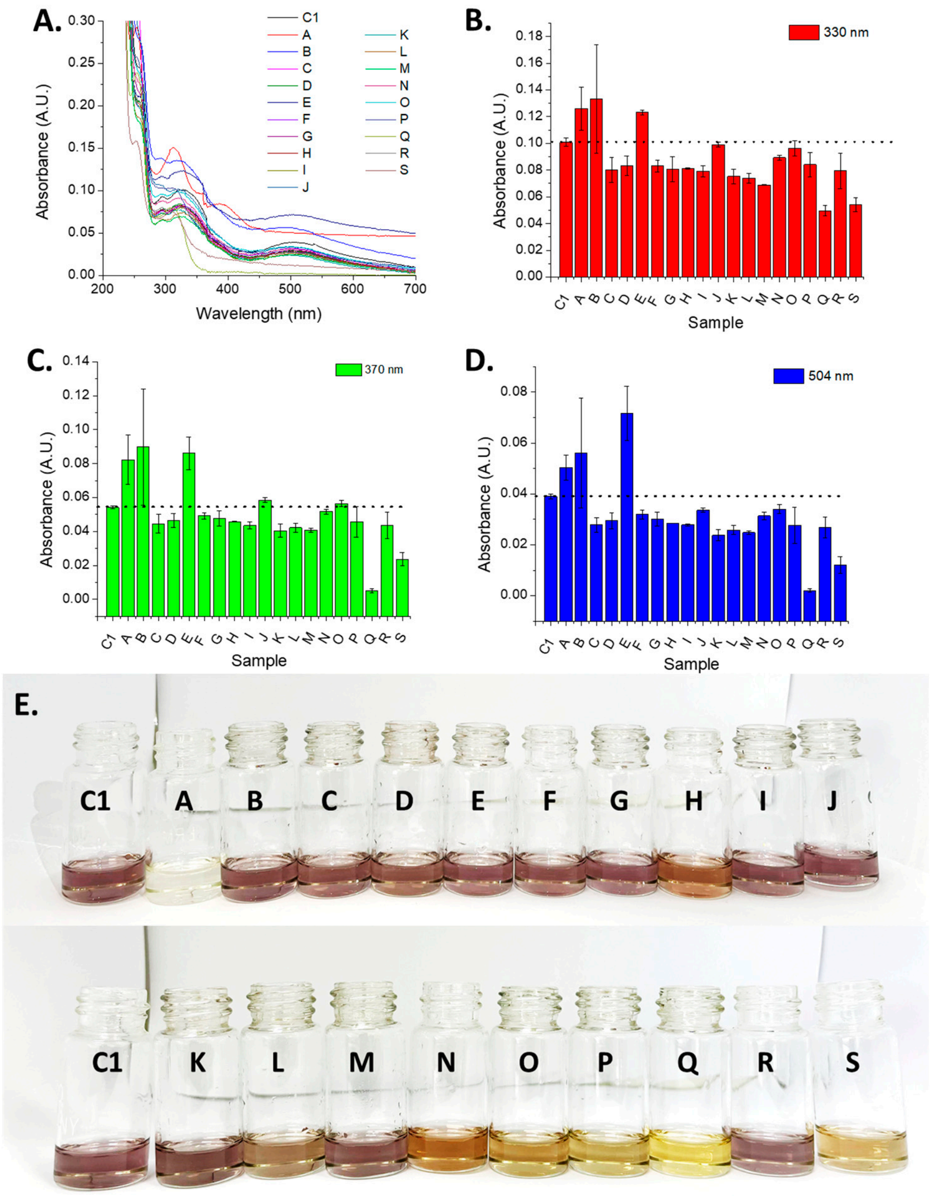

Figure S4: Spectral and color change of the iron (III) complex after the interaction with several analytes. **A.** Molecular absorption spectrum of iron (III)-complex (**C1**) and their interaction with several analytes. **B.** Variation of absorbance at λ= 330 nm. **C.** Variation of absorbance at λ= 370 nm. **D.** Variation of absorbance at λ= 504 nm. **E.** Naked eye color change of the iron (III) complex with all analytes assessed. **Sample code: A.** Hydroxide (OH^{-1}), **B.** Metabisulfite ($S_2O_5^{-2}$), **C.** Iodide (I^{-1}), **D.** Acetate ($MeCO_2^{-1}$), **E.** Nitrate (NO_3^{-1}), **F.** Sulfate (SO_4^{-2}), **G.** Thiosulfate ($S_2O_3^{-2}$), **H.** Bicarbonate (HCO_3^{-1}), **I.** Bisulfite (HSO_3^{-1}), **J.** Iodate (IO_3^{-1}), **K.** Cyanide (CN^{-1}), **L.** Fluoride (F^{-1}), **M.** Sulfide (S^{-2}), **N.** Carbonate (CO_3^{-2}), **O.** Citrate ($C_6H_5O_7^{-3}$), **P.** Monoacid phosphate (HPO_4^{-2}), **Q.** Diacid phosphate ($H_2PO_4^{-1}$), **R.** Arsenite (AsO_2^{-1}), **S.** Tartrate ($C_4H_4O_6^{-2}$). C_{C1}= 20 μM, C_{Sample}= 10 mol equivalents, Solvent= ACN.

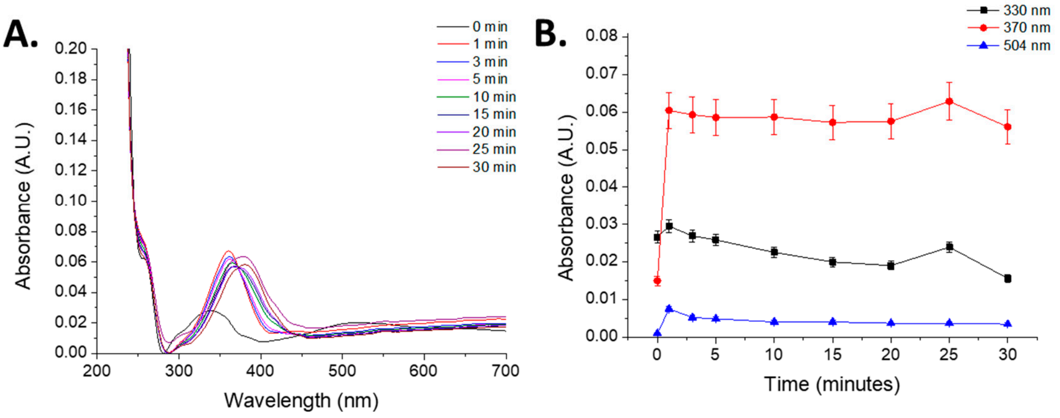

Figure S5: Kinetic profile of the interaction between the iron (III)-complex (**C1**) and NaOH. **A.** Overlapping spectra at different times. **B.** Variation of the main wavelength in the time. C_{C1}= 20 μM, C_{NaOH}= 10 mol equivalents, Solvent= ACN.

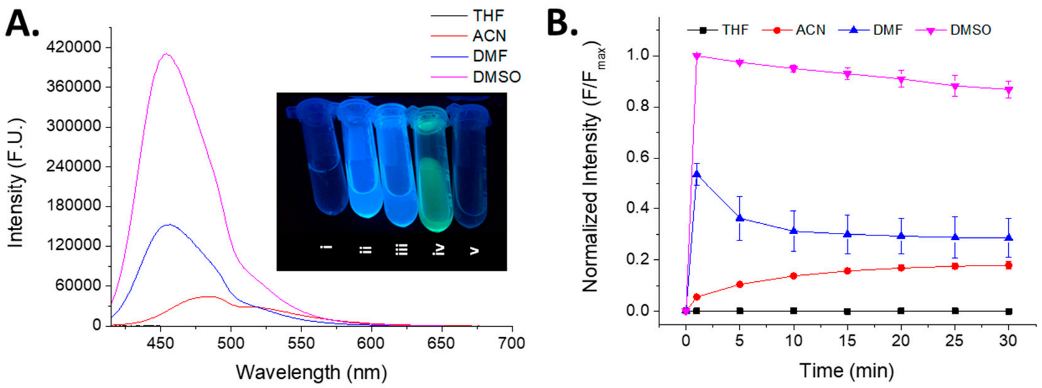

Figure S6: Kinetic profile of the interaction between the iron (III)-complex (**C1**) and NaOH using different solvents. **A.** Overlapping spectra at different times and naked-eye emission color changes. **B.** Variation of maximum emission wavelength in time. C_{C1}= 20 μM, C_{NaOH}= 10 mol equivalents, Incubation time: 3 min. **Image code: i:** iron (III)-complex dissolved in DMSO. **ii**: iron (III)-complex solved in ACN and 10 molar equivalents of NaOH. **iii**: iron (III)-complex dissolved in DMF and 10 molar equivalents of NaOH. **iv**: iron (III)-complex solved in DMSO and 10 molar equivalents of NaOH. **v**: iron (III)-complex solved in THF and 10 molar equivalents of NaOH.

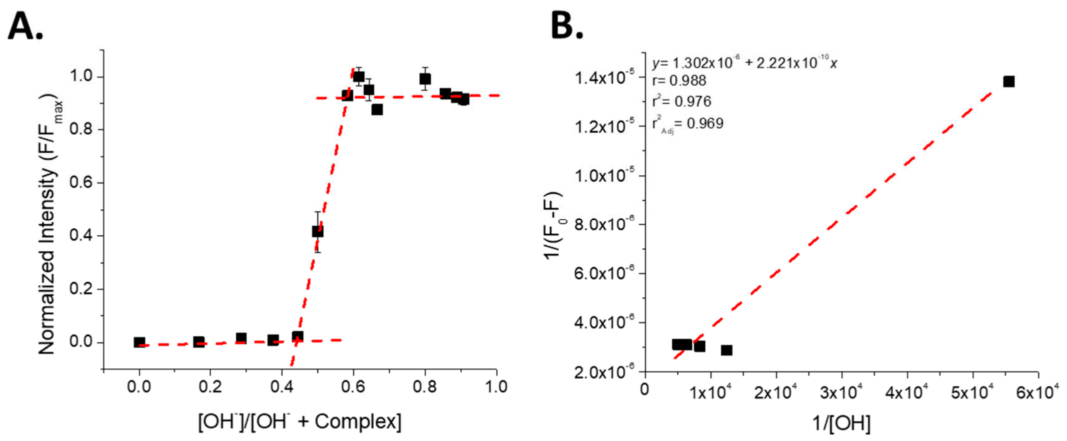

Figure S7: Analytical graphs from titration of the iron (III)-complex (**C1**). **A.** Job plot to determine the stoichiometric relationship between the iron (III)-complex (**C1**) and NaOH using the emission change and the molar fraction of the complex. **B.** Benesi-Hildebrand plot to determine the association constant to stoichiometric 1:1. C_{C1}= 20 μM, λ_{Ex}= 399 nm; λ_{Em}= 456 nm; solvent = DMSO; incubation time = 3 min.

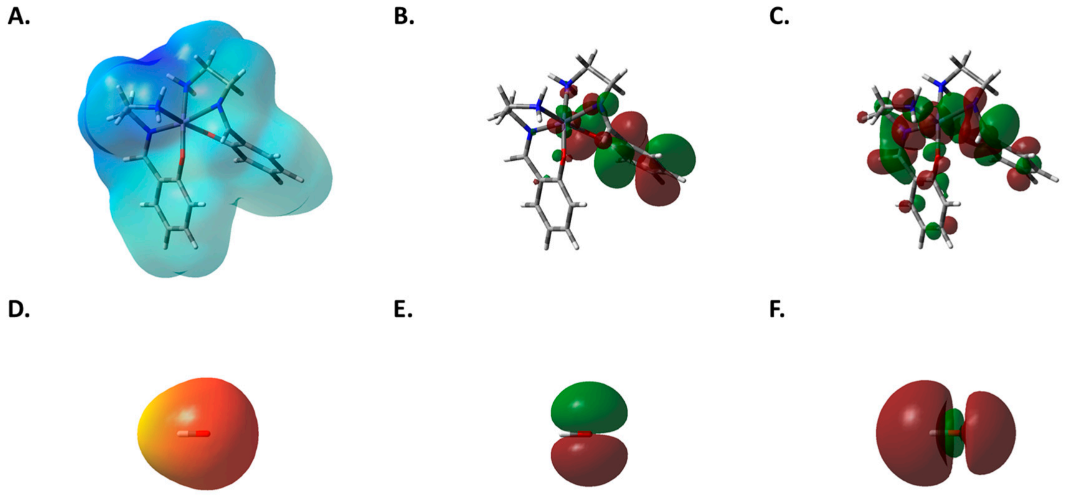

Figure S8: Electrostatic potential map (ESP) and molecular orbitals of the iron (III)-complex (**C1**) and the hydroxyl anion. **A.** ESP of the complex (**C1**). **B.** HOMO of the complex (**C1**). **C.** LUMO of the complex (**C1**). **D.** ESP of the hydroxyl anion. **E.** HOMO of the hydroxyl anion. **F.** LUMO of the hydroxyl anion.

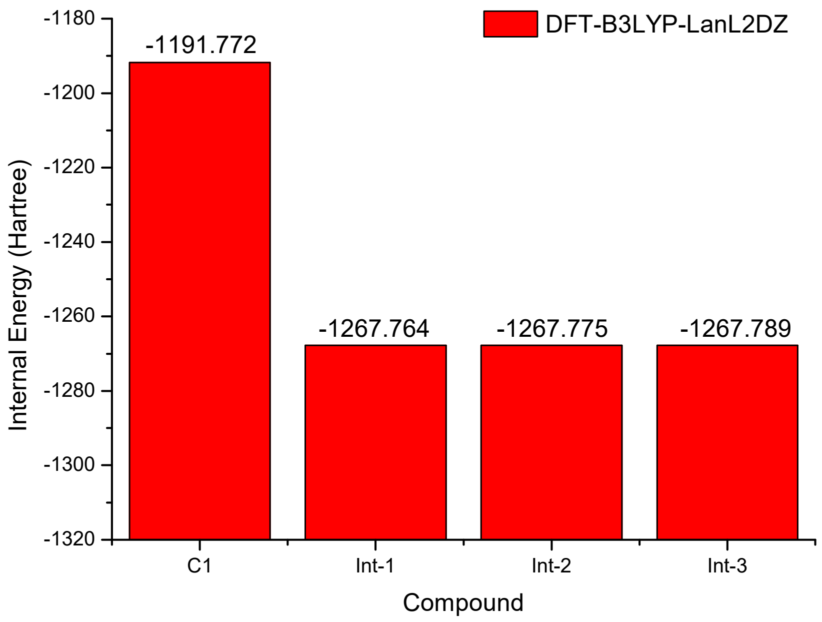

Figure S9: Internal energies of the iron (III)-complex (**C1**) and the intermediates proposed in the reaction mechanism.

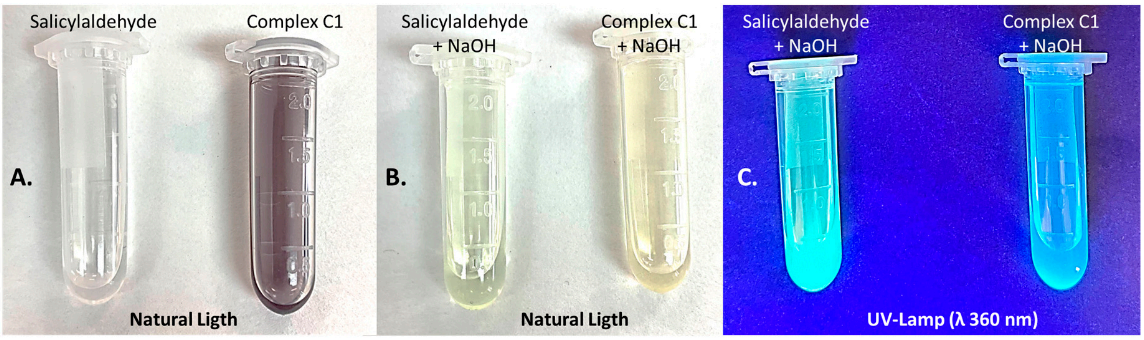

Figure S10: Comparison of the colorimetric changes between salicylaldehyde and the iron (III)-complex (**C1**) after and later the addition of NaOH dissolution.

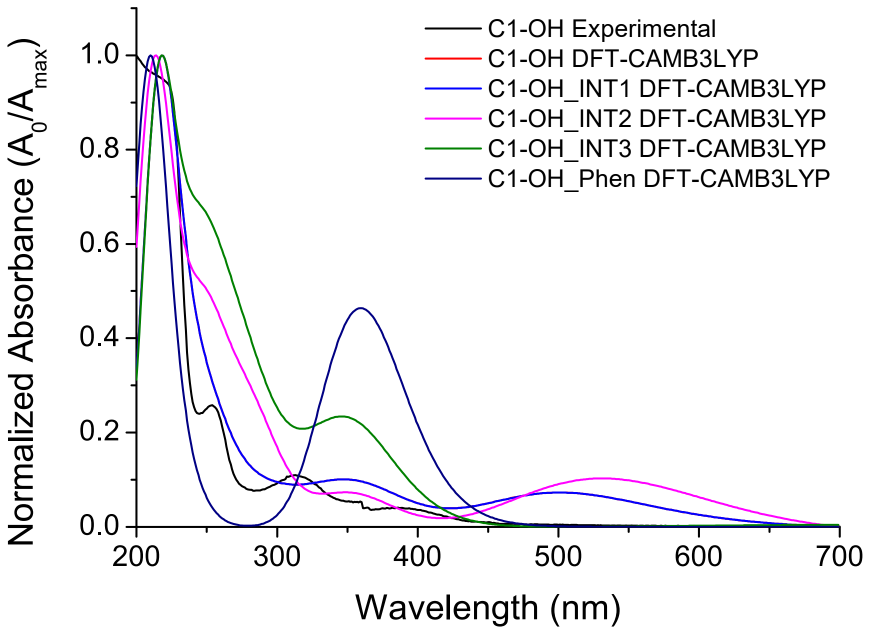

Figure S11: Overlapping experimental UV-Vis spectrum and all intermediates proposed in the reaction mechanism.

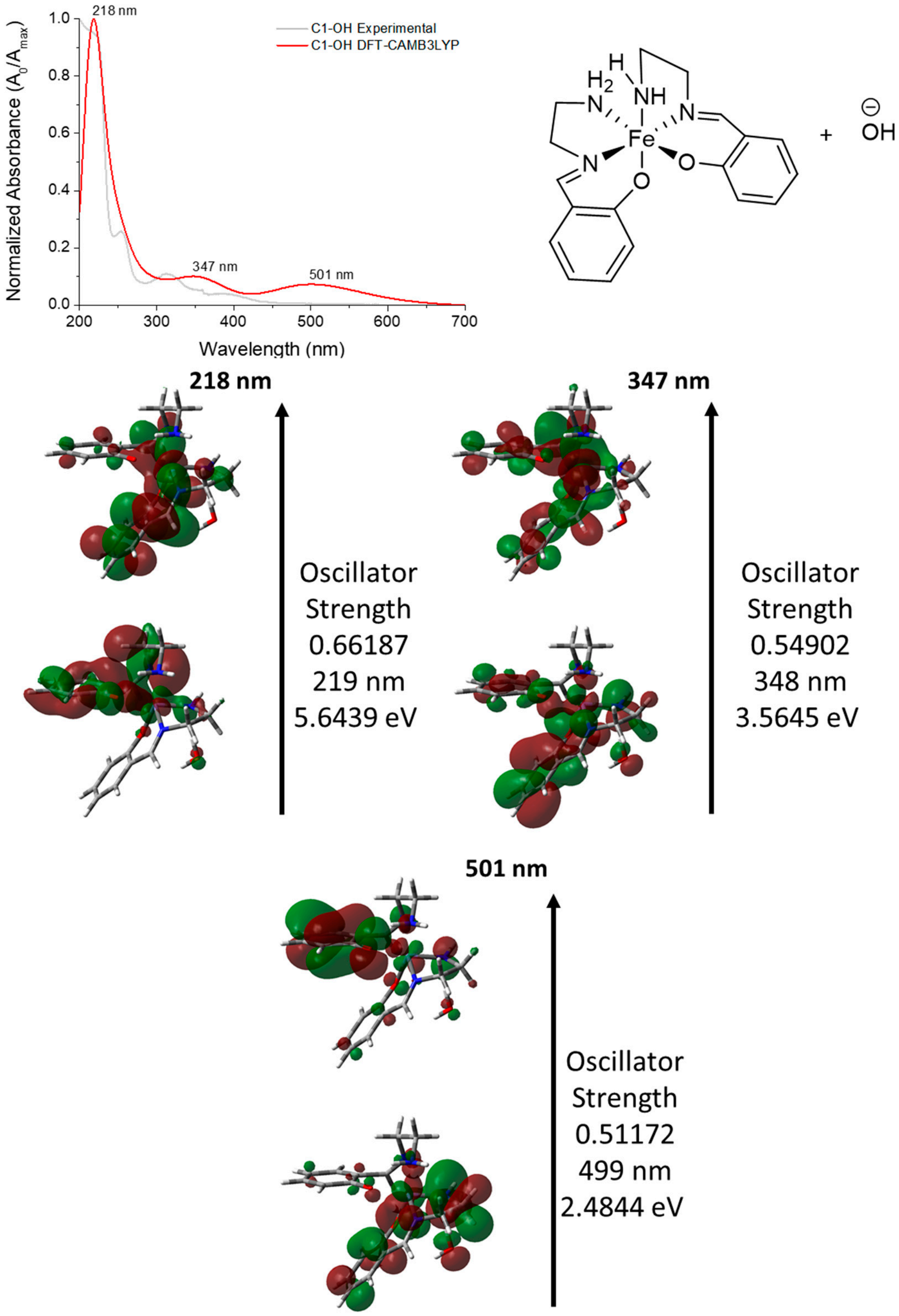

Figure S12: Experimental UV-Vis spectra of complex C1 + OH- and their calculated spectra

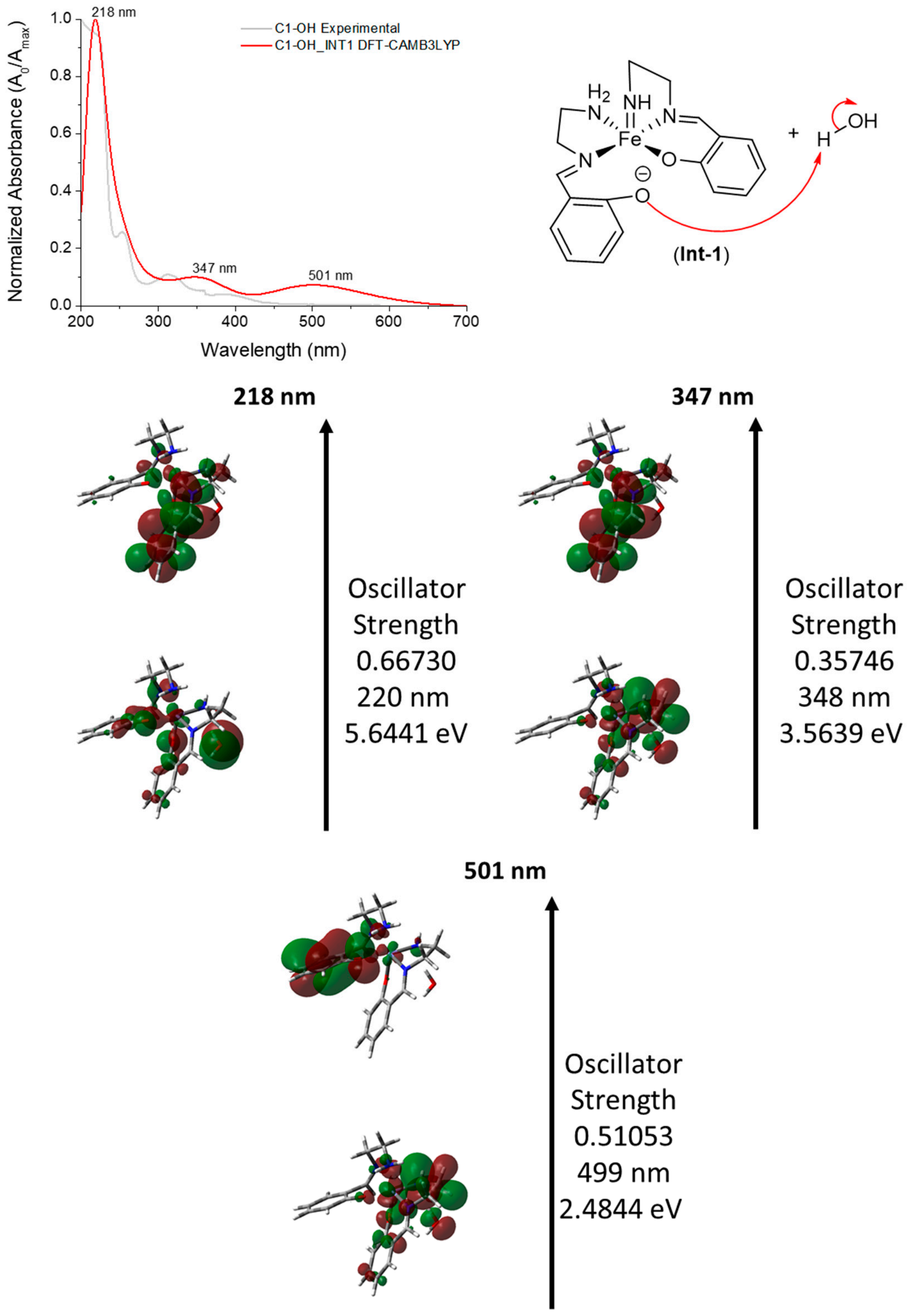

Figure S13: Experimental UV-Vis spectra of complex C1 + OH- and the calculated spectra of proposal intermediate Int-1 together to each electronic transition.

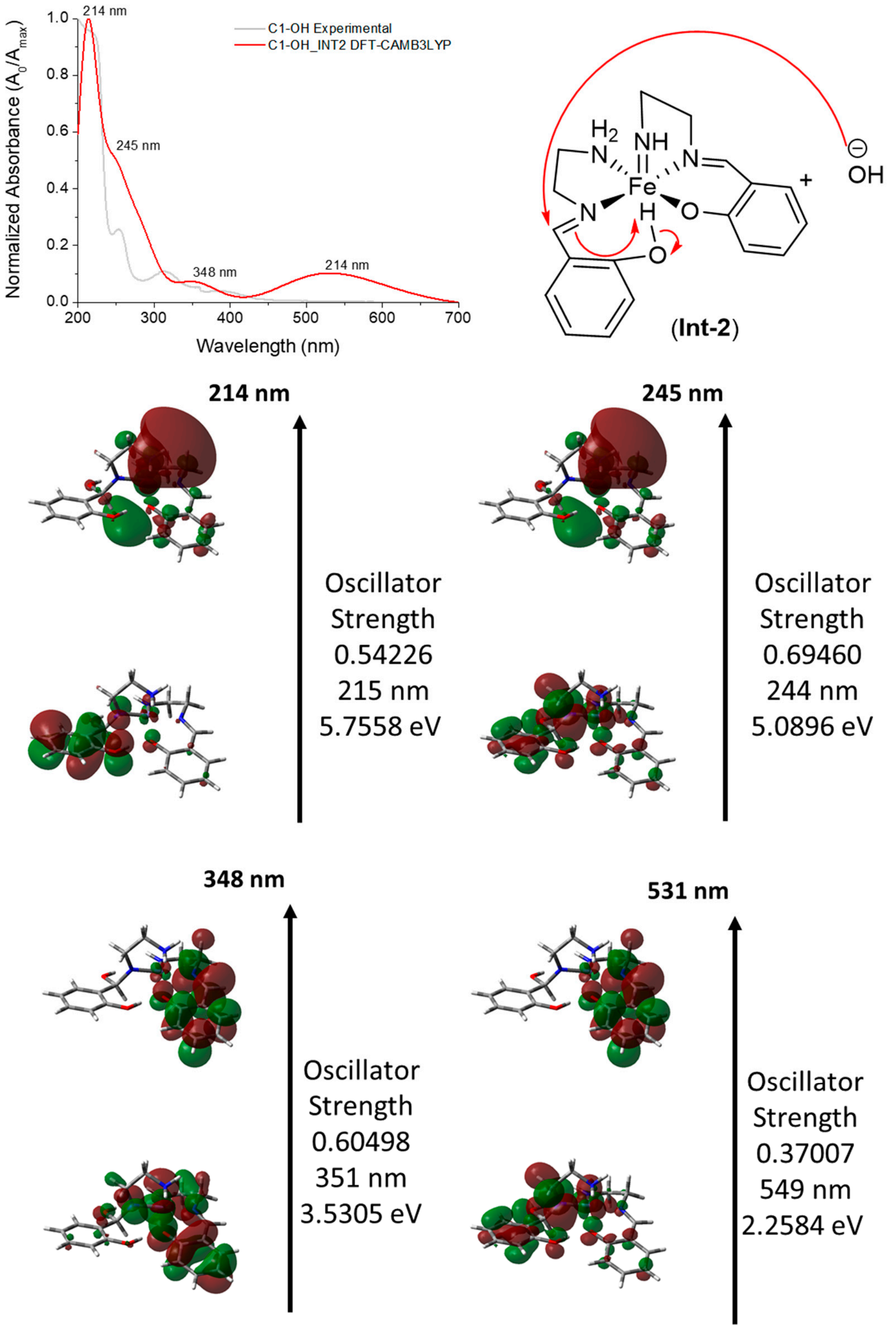

Figure S14: Experimental UV-Vis spectra of complex C1 + OH- and the calculated spectra of proposal intermediate Int-2 together to each electronic transition.

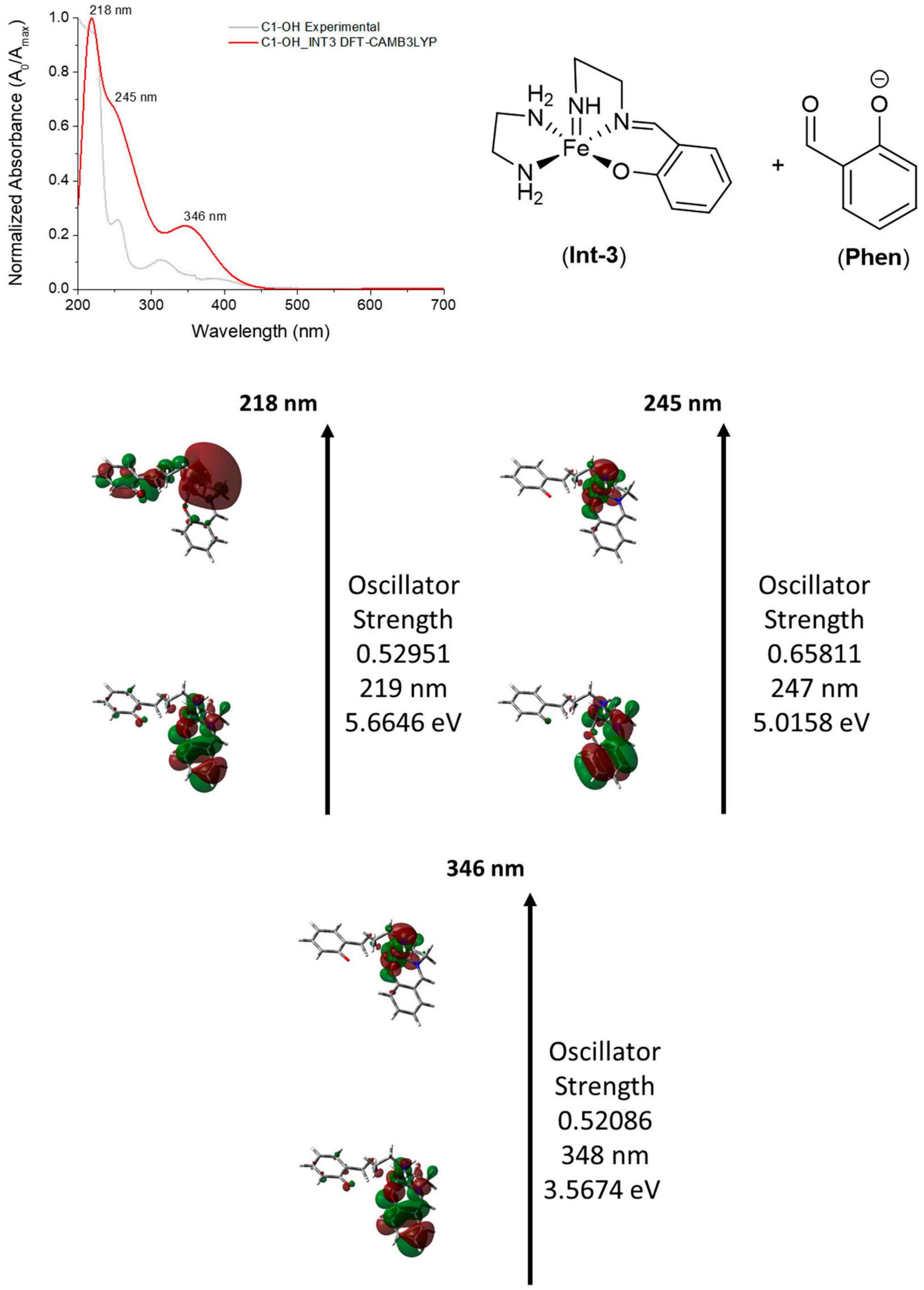

Figure S15: Experimental UV-Vis spectra of complex C1 + OH- and the calculated spectra of proposal intermediate Int-3 together to each electronic transition.

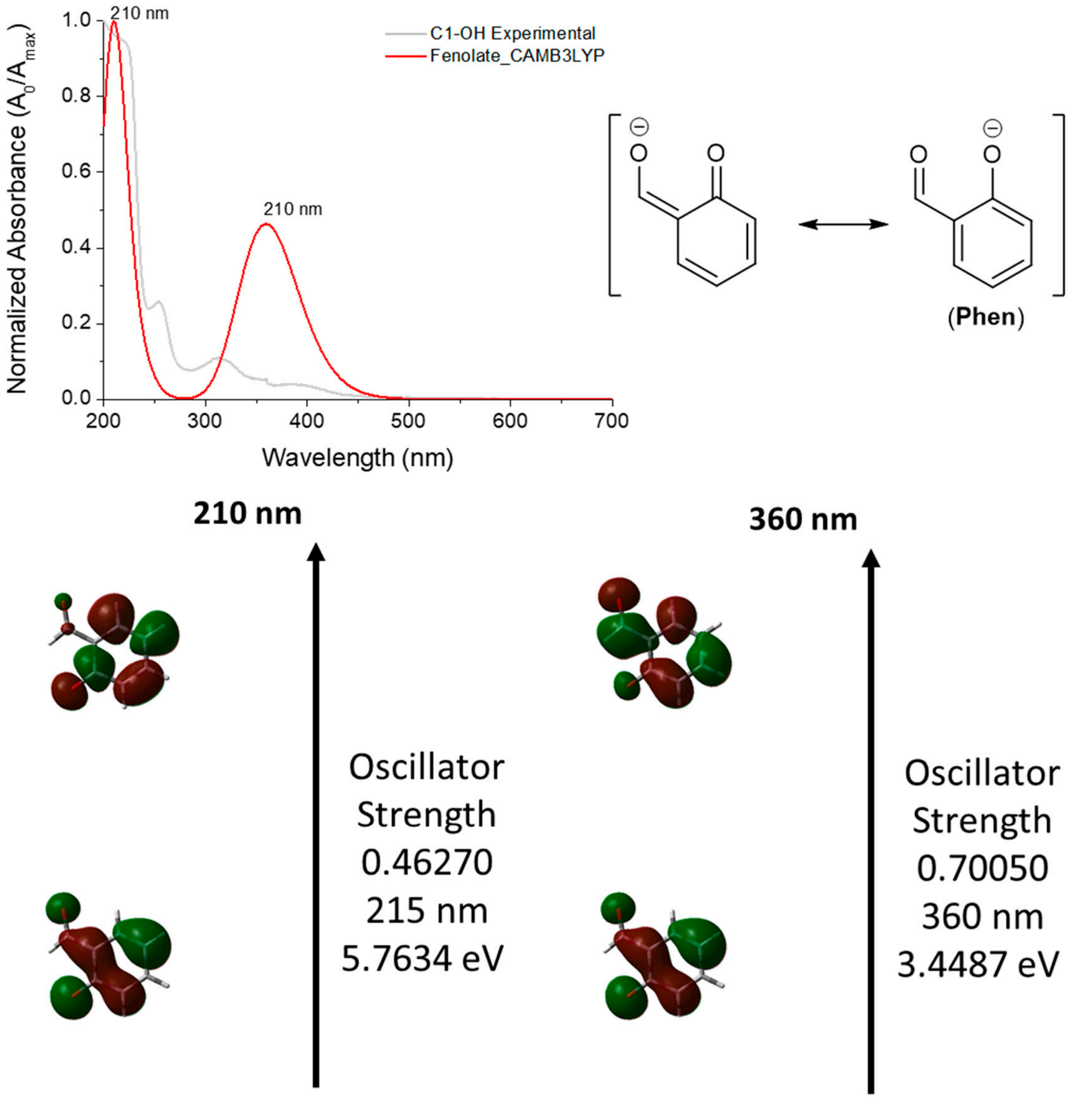

Figure S16: Experimental UV-Vis spectra of complex C1 + OH- and the calculated spectra of proposal intermediate phenolate together to each electronic transition.

3. Supplementary Tables

Table S1: Crystal data and details of structure refinement of complex C1.

Formula	$C_{18}H_{22}BrFeN_4O_2$
Formula weight (*gmol*$^{-1}$)	462.15
Crystal system	Triclinic
Space group	P-1
a (Å)	9.3079(9)
b (Å)	9.4322(9)
c (Å)	10.6985(9)
α (°)	90.652(3)
β (°)	92.762(4)
γ (°)	98.425(4)
V (Å^3)	927.89(15)
Z	2
D_{calc} (*gcm*$^{-3}$)	1.654
μ (mm-1)	2.986
F(000)	470
R_{int}	0,0323
Total reflections	68598
Radiation	MoKα (λ = 0.71073)
$I > 2\sigma(I)$	4108
R_1, wR_2 $I > 2\sigma(I)$	0.0253, 0.0630
T (K)	170
Maximum, minimum electron density (e/Å^3)	0.40/-0.63

Table S2: Bond distances and selected angles in the metal coordination sphere of C1 at 170 K.

Bond length (Å)			
Fe1-O2	1.19192(15)	Fe1-N4	2.1921(17)
Fe1-N3	2.1117(17)	Fe1-N2	2.2040(18)
F1-N1	2.1032(16)	Fe1-O1	1.9205(15)
**Bond angle (°)**			
O2-Fe1-N3	86.95(6)	N1-Fe1-N2	77.37(6)
O2-Fe1-N1	101.74(6)	N4-Fe1-N2	85.00(7)
O2-Fe1-N4	161.95(6)	O1-Fe1-N3	95.52(6)
O2-Fe1-N2	89.01(7)	O1-Fe1-N1	86.26(6)
O2-Fe1-O1	100.76(7)	O1-Fe1-N4	89.66(7)
N3-Fe1-N4	77.33(6)	O1-Fe1-N2	162.37(6)
N3-Fe1-N2	99.66(6)	Σ/°	79.6
N1-Fe1-N3	170.69(7)	Θ/°	226.1
N1-Fe1-N4	93.56(6)	α/°	100.8

Table S3: Cartesian Coordinates of each atom from iron (III) complex (C1)

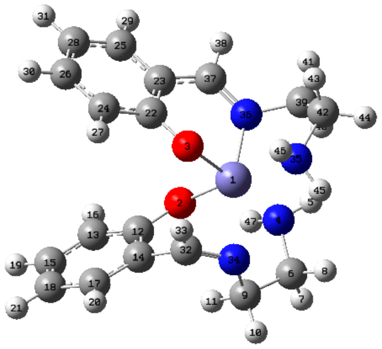

Center Number	Atomic Number	Atomic Type	Coordinates (Angstroms)		
			X	Y	Z
1	26	0	1.151449	-0.143034	0.095905
2	8	0	-0.20656	-0.636271	-1.101656
3	8	0	-0.022426	0.798051	1.226841
4	7	0	2.307929	-1.257847	-1.21083
5	1	0	3.190967	-0.848494	-1.520992
6	6	0	2.517189	-2.668285	-0.72907
7	1	0	2.775222	-3.335933	-1.560823
8	1	0	3.352413	-2.666339	-0.019491
9	6	0	1.245024	-3.13797	-0.026393
10	1	0	1.429633	-4.127152	0.412233
11	1	0	0.412401	-3.232398	-0.734358
12	6	0	-1.359982	-1.293471	-0.726267
13	6	0	-2.474145	-1.287191	-1.590906
14	6	0	-1.453197	-1.966497	0.53139
15	6	0	-3.663009	-1.929836	-1.20833
16	1	0	-2.393537	-0.765858	-2.540028
17	6	0	-2.668216	-2.567641	0.919941
18	6	0	-3.768658	-2.568109	0.047009
19	1	0	-4.515734	-1.920237	-1.882293
20	1	0	-2.743855	-3.052654	1.890842
21	1	0	-4.696467	-3.05075	0.338877
22	6	0	-0.87645	1.722041	0.724557
23	6	0	-0.535211	2.54854	-0.404598
24	6	0	-2.121977	1.930693	1.369461
25	6	0	-1.456079	3.538081	-0.858119
26	6	0	-3.016028	2.889427	0.886296
27	1	0	-2.363879	1.319084	2.232927
28	6	0	-2.691899	3.696873	-0.237769
29	1	0	-1.18256	4.169113	-1.701576
30	1	0	-3.974731	3.021548	1.381378

31	1	0	-3.398274	4.439008	-0.596149
32	6	0	-0.268271	-1.874549	1.416202
33	1	0	-0.398101	-1.372137	2.375473
34	7	0	0.969372	-2.215613	1.113137
35	7	0	2.751816	0.26651	1.282523
36	7	0	1.646079	1.516821	-0.725744
37	6	0	0.80479	2.513366	-0.919253
38	1	0	1.175758	3.411803	-1.421737
39	6	0	3.108516	1.733641	-0.716752
40	1	0	3.610028	1.076023	-1.434597
41	1	0	3.369069	2.765532	-0.9834
42	6	0	3.55831	1.427117	0.739943
43	1	0	3.360556	2.301031	1.36751
44	1	0	4.631274	1.212657	0.786221
45	1	0	3.296448	-0.59002	1.402358
46	1	0	2.315381	0.49806	2.179471
47	1	0	1.631565	-1.233727	-1.98452

Table S4: Cartesian Coordinates of each atom from iron (III) complex (C1) with analyte OH^-

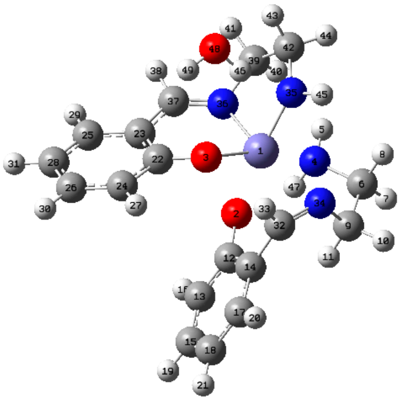

Center Number	Atomic Number	Atomic Type	Coordinates (Angstroms)		
			X	Y	Z
1	26	0	0.906591	-0.686285	-0.000385
2	8	0	-0.71286	-0.56668	-1.14219
3	8	0	0.1817	0.745654	1.050685
4	7	0	1.232547	-2.22246	-1.313531
5	1	0	2.174391	-2.214446	-1.702922
6	6	0	0.900125	-3.559522	-0.731622
7	1	0	0.813372	-4.326725	-1.514638
8	1	0	1.712825	-3.837384	-0.052333
9	6	0	-0.40386	-3.447809	0.060424
10	1	0	-0.613271	-4.42343	0.520492
11	1	0	-1.245915	-3.195393	-0.598614
12	6	0	-1.984333	-0.662459	-0.675628
13	6	0	-3.072713	-0.220447	-1.471872
14	6	0	-2.270455	-1.182234	0.630009
15	6	0	-4.386822	-0.275906	-0.985007
16	1	0	-2.852494	0.180324	-2.457718
17	6	0	-3.59176	-1.18798	1.121371
18	6	0	-4.658706	-0.754062	0.316748
19	1	0	-5.204298	0.071463	-1.614007
20	1	0	-3.785182	-1.551126	2.129908
21	1	0	-5.677602	-0.779437	0.69368
22	6	0	-0.13464	1.936085	0.511836
23	6	0	0.572037	2.501979	-0.604818
24	6	0	-1.178013	2.704314	1.1018
25	6	0	0.22615	3.796209	-1.079924
26	6	0	-1.515045	3.963262	0.598924
27	1	0	-1.709403	2.272789	1.945094
28	6	0	-0.815741	4.522489	-0.502268
29	1	0	0.788682	4.217824	-1.912269

30	1	0	-2.325691	4.522749	1.061249
31	1	0	-1.083119	5.504874	-0.881003
32	6	0	-1.109215	-1.588394	1.464128
33	1	0	-0.935774	-1.02715	2.383255
34	7	0	-0.19756	-2.487608	1.174466
35	7	0	2.499801	-1.011762	0.87655
36	7	0	2.0028	0.558539	-0.975578
37	6	0	1.733472	1.827764	-1.137588
38	1	0	2.475339	2.451521	-1.647334
e	6	0	3.378146	0.039416	-1.161511
40	1	0	3.395849	-0.663078	-2.006356
41	1	0	4.079913	0.85055	-1.39296
42	6	0	3.760443	-0.674669	0.173946
43	1	0	4.369306	0.006246	0.786683
44	1	0	4.376307	-1.562441	-0.046359
45	1	0	2.563318	-1.765392	1.562414
46	1	0	2.52643	0.522476	1.92465
47	1	0	0.536969	-1.92151	-2.008662
48	8	0	2.846213	1.35979	2.361859
e	1	0	2.07073	1.914834	2.567882

Please contact corresponding author (Prof. Dr. Marco Mellado, marco.mellado@ucentral.cl) if you need any additional output file of the computational calculus.
